# Bioactivity of Marine-Derived Peptides and Proteins: A Review

**DOI:** 10.3390/md23040157

**Published:** 2025-04-04

**Authors:** Fereidoon Shahidi, Abu Saeid

**Affiliations:** Department of Biochemistry, Memorial University of Newfoundland, St. John’s, NL A1C 5S7, Canada; asaeid@mun.ca

**Keywords:** marine organism, protein hydrolysates, peptides, bioactivity, functional foods

## Abstract

The marine environment, covering over 70% of the Earth’s surface, serves as a reservoir of bioactive molecules, including peptides and proteins. Due to the unique and often extreme marine conditions, these molecules exhibit distinctive structural features and diverse functional properties, making them promising candidates for therapeutic applications. Marine-derived bioactive peptides, typically consisting of 3 to 40 amino acid residues—though most commonly, 2 to 20—are obtained from parent proteins through chemical or enzymatic hydrolysis, microbial fermentation, or gastrointestinal digestion. Like peptides, protein hydrolysates from collagen, a dominant protein of such materials, play an important role. Peptide bioactivities include antioxidant, antihypertensive, antidiabetic, antimicrobial, anti-inflammatory, anticoagulant, and anti-cancer effects as well as immunoregulatory and wound-healing activities. These peptides exert their effects through mechanisms such as enzyme inhibition, receptor modulation, and free radical scavenging, among others. Fish, algae, mollusks, crustaceans, microbes, invertebrates, and marine by-products such as skin, bones, and viscera are some of the key marine sources of bioactive proteins and peptides. The advancements in the extraction and purification processes, e.g., enzymatic hydrolysis, ultrafiltration, ion-exchange chromatography, high-performance liquid chromatography (HPLC), and molecular docking, facilitate easy identification and purification of such bioactive peptides in greater purity and activity. Despite their colossal potential, their production, scale-up, stability, and bioavailability are yet to be enhanced for industrial applications. Additional work needs to be carried out for optimal extraction processes, to unravel the mechanisms of action, and to discover novel marine sources. This review emphasizes the enormous scope of marine-derived peptides and proteins in the pharmaceutical, nutraceutical, cosmeceutical, and functional food industries, emphasizing their role in health promotion and risk reduction of chronic diseases.

## 1. Introduction

Marine environments comprise more than 70% of the Earth’s surface, an unparalleled biodiversity reservoir. These ecosystems host an extraordinary combination of organisms, ranging from microscopic phytoplankton to massive marine mammals, all of which have evolved to thrive under unique and often extreme conditions. These adaptations have been directed to producing bioactive molecules, including peptides and proteins, with distinct structural features, diversity, and functional properties compared with substances isolated from terrestrial plants [1,2].

Proteins are vital macronutrients that act as a primary energy source and provide essential amino acids for regular physiological functions such as growth, development, and maintenance. Bioactive peptides (short amino acid sequences) are protein fragments thought to contribute to various functional and physiological properties, playing a crucial role in health and metabolism [1]. Marine-derived bioactive peptides typically consist of 3 to 40 (mostly 2–20) amino acid residues. These peptides are inactive within their parent protein sequences but become biologically active when released through gastrointestinal digestion, enzymatic processing, or fermentation. Depending on the amino acid sequence, composition, and molecular weight, they are involved in various biological functions. They interact with cellular receptors, enzymes, and ion channels to modulate physiological processes. Marine-derived peptides induce cell death through various mechanisms, including apoptosis, modulation of tubulin microtubule balance (anti-microtubule activity), vascular inhibition, anti-proliferative effects, and cytotoxicity [3,4,5]. The primary marine organisms such as fish, seaweed, shellfish, microalgae, crustaceans, cephalopods, and mollusks and their by-products are promising sources of proteins and bioactive peptides. These proteins and peptides from marine organisms have gained significant attention due to their diverse bioactive potential, such as antioxidant, antihypertensive, antidiabetic, anticoagulant, antimicrobial, anti-inflammatory, anti-cancer, and immunoregulatory effects, among others. Their multifunctional properties hold immense potential for applications in the food, pharmaceutical, and cosmeceutical industries [1,6,7,8].

What makes marine-derived peptides and proteins especially compelling is their structural uniqueness, driven by the environmental pressures of their origin. Unlike their terrestrial counterparts, these molecules are often characterized by high stability, bioavailability, sustainability, and low toxicity. Marine organisms face high salinity, extreme pressure, variable oxygen levels, and fluctuating temperatures. These factors have shaped their biochemical adaptations, resulting in the synthesis of compounds with superior functionality [1,9].

Furthermore, advances in extraction and analytical techniques have facilitated the identification and characterization of bioactive peptides and proteins from marine sources. Bioactive peptides can be produced from protein by hydrolysis using conventional methods or advanced methods, including acid or alkaline, solvent extraction, enzymatic hydrolysis, microbial fermentation, ultrasound, pulsed electric field, pressurized liquid, and subcritical water extraction techniques [10]. Compared with other methods, the enzymatic hydrolysis method is preferred in the food and pharmaceutical industries because the other methods can leave residual organic solvents or toxic chemicals in the final products [11]. Marine-derived peptides, obtained through protein degradation methods, often contain impurities that can hinder their functionality and effectiveness. Advanced separation and purification techniques are essential to enhance these bioactive compounds’ physical activity and purity to harness their full potential. Purification is a critical step, especially when preparing peptides for consumer applications, as it ensures the desired level of quality and effectiveness. State-of-the-art methods such as ultrafiltration, gel filtration chromatography, ion-exchange chromatography, and reversed-phase high-performance liquid chromatography (RP-HPLC) are commonly employed to achieve this. For instance, immunoregulatory peptides derived from *Stolephorus chinensis* were successfully isolated through process optimization, ultrafiltration, ion-exchange chromatography, and RP-HPLC. These advanced techniques not only improve the purity of the peptides but also maximize their bioactive properties, paving the way for their application in pharmaceuticals, nutraceuticals, and functional foods [12,13,14,15].

This review delves into the bioactive potential of marine-derived peptides and proteins, emphasizing their sources, key bioactivities, mechanisms of action, and applications across various sectors. It also addresses the challenges in their extraction and utilization while highlighting future research directions for maximizing their benefits.

## 2. Marine-Derived Peptides and Proteins: Sources and Characteristics

**Figure 1:**
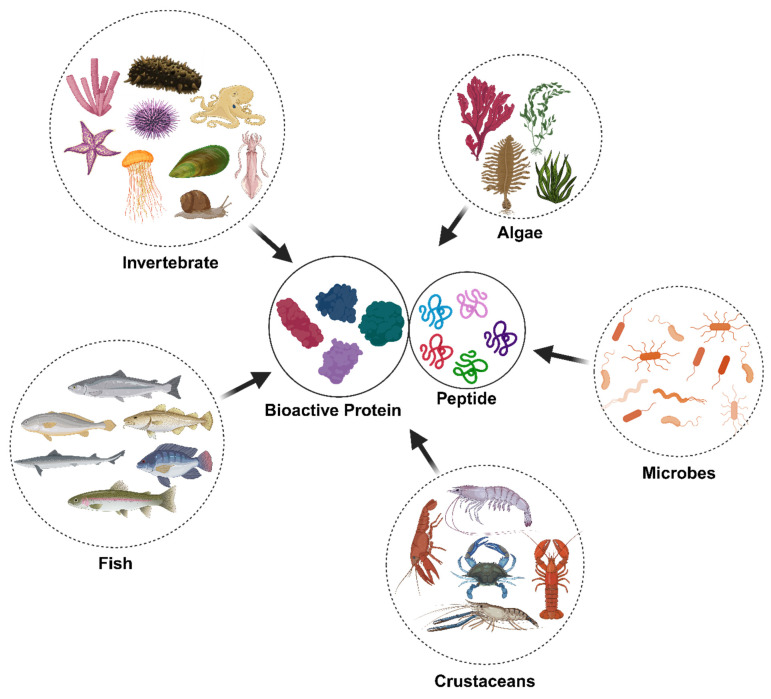
Major groups of marine organisms as sources of bioactive proteins and peptides.

Despite the diversity of the marine ecosystem, studies on their bioactive proteins and peptides have been carried out on only a few organisms, such as some invertebrates (i.e. mollusks, sponges, echinoderms, cnidarians, etc.), fishes, algae (i.e. red, green, and brown algae, spirulina, etc.), crustaceans (i.e. crabs, crayfish, lobster, prawns, and shrimp), and marine microbes (Figure 1). Fishes, as leading sources of high-quality protein, have essential amino acids for human metabolism. Fish protein consists of various structural and functional proteins including collagen, myoglobin, and actins, which are abundantly distributed in tissues such as skin, bones, scales, and internal organs. Marine-derived proteins and peptides from fishes include fillets, discards, and coproducts [16,17,18,19]. In addition to fish, collagens have been extracted from other marine sources, including sea cucumbers, mollusks, sponges, crustaceans, seaweed, and jellyfish [20,21]. Shellfish comprises shrimp, crab, crayfish, and lobster and is a good source of proteins and peptides. Bioactive peptides in shellfish include the opioid peptides standard in mussels and clams; antimicrobial peptides in blue mussels, oysters, scallops, and shrimp; angiotensin-converting enzyme (ACE) inhibitory peptides in shrimp and crabs; immunomodulatory peptides found in fish, mussels, and scallops; and antioxidant peptides in scallops. Opioid peptides in shellfish include dynorphins in blue mussels (*Mytilus edulis*), methionine-enkephalins in oysters and clams, and β-endorphins in shrimp [22,23,24,25]. Shellfish-derived ACE inhibitory peptides play a role in lowering blood pressure by inhibiting ACE activity. Notable examples include valyl-tyrosine (VY) from protein hydrolysates of shrimp (*Litopenaeus vannamei*), isoleucyl-prolyl-proline from oysters (*Crassostrea gigas*), isoleucyl-leucine-proline from protein hydrolysates of crabs (*Portunus trituberculatus*), and leucine-aspartic acid from protein hydrolysates of clams (*Meretrix meretrix*). Additionally, shellfish contain immunomodulatory proteins and peptides, such as hemocyanins in mollusks and crustin in crustaceans, which enhance phagocytosis and promote cytokine production [26]. Studies with spontaneously hypertensive rats have reported that gelatin hydrolysates from sea cucumber, skate skin, jellyfish, and squid skin can reduce blood pressure, a controllable risk factor in the development of cardiovascular diseases. Fish gelatin hydrolysates and peptides demonstrate diverse biological activities, including antioxidant, anti-anemia, immunoregulatory, calcium binding, mineral chelating, ACE and DPP-IV inhibitory, antimicrobial, and antihypertensive properties. These bioactivities have been identified in hydrolysates and peptides derived from gelatin extracted from various fish species, such as Atlantic salmon, cod, herring, hoki, Pacific whiting, pollack, snapper, and sole [27,28]. Researchers have successfully isolated antimicrobial peptides (AMPs) from various marine organisms, including Atlantic cod (*Gadus morhua*), mud crab (*Scylla paramamosain*), oyster (*Crassostrea gigas*), yellow catfish (*Pelteobagrus fulvidraco*), sponge (*Trichoderma* sp.), marine snail (*Cenchritis muricatus*), marine bivalve (*Mytilus coruscus*), and octopus (*Octopus vulgaris*). These AMPs are characterized by their safety, natural origin, cost-effectiveness, and high bioactivity, making them promising candidates for various applications [29,30]. Furthermore, AMPs have been identified in various mollusk species, including bivalves. Cysteine-rich peptides were specifically characterized in mussels, while defensins and proline-rich peptides have been detected in both oysters and gastropods. Additionally, proteins exhibiting antimicrobial properties, such as egg case proteins (Sep-ECPs) and hemocyanin, have been isolated from gastropods [31]. Recent investigations have increasingly focused on isolating bioactive peptides with potential anti-cancer properties from various marine organisms, including sponges, tunicates, ascidians, mollusks, fish and their by-products, and other aquatic species [9,32,33,34]. Studies indicate that marine organisms serve as valuable sources of anticoagulant peptides, with algae, starfish, mussels, and marine echinuroid worms being the most common [35]. Marine microalgae are a rich source of antioxidant peptides, such as glutathione and carnosine, which are commonly associated with animal muscles [36]. Key functional algal proteins include phycobiliproteins and lectins. Phycobiliproteins such as phycocyanins and phycoerythrin function as fluorescent markers in biotechnology and natural colorants in food and cosmetics while exhibiting antioxidant, anti-inflammatory, and antiviral properties. Macroalgal lectins in species like *Ulva* sp. display antibacterial, antiviral, antitumor, and anti-inflammatory activities through carbohydrate-binding interactions [37]. These bioactive compounds hold significant potential for pharmaceutical and nutraceutical applications.

## 3. Production of Peptides from Marine Organisms

**Figure 2:**
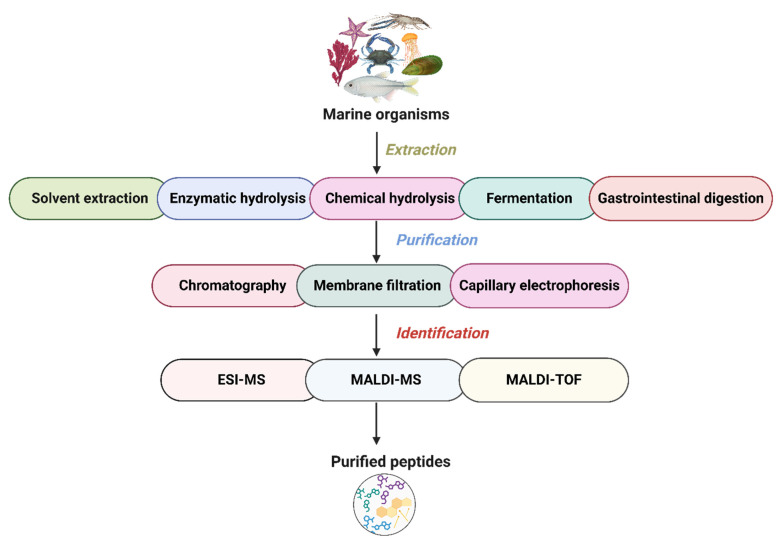
Production of bioactive peptides from marine sources.

The production of peptides from marine organisms begins with a pretreatment step followed by extraction via hydrolysis using chemical, fermentation, or enzymatic approaches. Subsequent purification is achieved through chromatography, membrane filtration, and capillary electrophoresis. Finally, peptide identification is performed using advanced mass spectrometric techniques (Figure 2). Pretreatment improves the efficiency of the interaction between the enzyme’s active site and the specific binding regions within the protein sequence. This is usually achieved using thermal denaturation, homogenization, dialysis, drying, and removing of heavy metals and degreasing. Thermal denaturation (e.g., boiling) sterilizes microorganisms and inactivates tissue enzymes, preventing interference from nonspecific enzymatic activity. In addition, protein denaturation exposes more reactive sites and softens the structural conformation. Homogenization increases the total surface area of proteins, enabling subsequent processing steps. Furthermore, removing fat using solvents such as ethanol, heavy metals using EDTA, and salts through dialysis helps preserve enzymatic activity and improve the efficiency of enzymatic hydrolysis [38,39,40]. The most common extraction method used to produce marine peptides is solvent extraction. Various solvents extract peptides based on solvent polarity and the peptide’s solubilizing ability. The common solvents used in marine peptide extraction are ethanol, methanol, acetone, ethyl acetate, hexane, and butanol. Most importantly, adding acid to the solvent enhances extraction efficacy of the peptides. For instance, marine bivalves have been a source of bioactive peptides, as demonstrated by Jayasanta et al. [41], who extracted these peptides using six different solvent systems listed earlier. Among these, methanol was identified as the most effective solvent for isolating antimicrobial peptides, which showed activity against 13 different pathogenic bacteria. Similarly, Sruthy et al. [42] isolated antimicrobial peptides from *Psenopsis cyanea* using a modified acetic acid–actone precipitation method. In pursuing environmentally friendly extraction methods, deep eutectic solvents (DESs) are emerging as a sustainable option for peptide isolation, gaining across-the-board recognition for biological applications. For example, Bai et al. [43] exhibited the remarkable potential of DESs in collagen peptide isolation from cod skins, reaching an impressive 96% efficiency and a complete 100% yield using a choline chloride–oxalic acid solvent system. These methods have limitations, as they are time-consuming and less effective. Hence, a novel, green, cost-effective method is needed to overcome this limitation for the extraction of peptides. Nowadays, innovative extraction techniques are being employed to improve peptide recovery from marine organisms. These include ultrasound-assisted extraction, microwave-assisted extraction, supercritical fluid extraction, pressurized solvent extraction, pulsed electric field extraction, and enzymatic-assisted extraction, all of which improve the efficiency of solvent-based peptide extraction [44,45]. For instance, microwave- and ultrasound-assisted extraction methods have successfully isolated collagen protein from European plaice skin [46]. Furthermore, a combination of supercritical fluid extraction and subcritical water hydrolysis has the potential to achieve a high yield of functional compounds, including peptides, from squid viscera [47]. Moreover, Lakmal et al. [48] reported the application of enzyme-assisted extraction for producing bioactive peptides from microalgae, including *Spirulina maxima*, *Dunaliella salina*, and *Rhodosorus* sp. They recommended this method as a practical approach for recovering novel bioactive peptides from microalgal sources.

Chemical methods represent conventional hydrolysis techniques using chemical agents to cleave protein molecules into peptide fragments. These agents can be either acidic, as in acid hydrolysis, or alkaline, as in alkali hydrolysis. Acid hydrolysis utilizes hydrochloric acid or sulfuric acid under elevated temperatures at 138 °C and pressure at 310 MPa for several hours to facilitate protein hydrolysis. The resulting mixture is then neutralized and dehydrated; however, this process generates a high salt concentration, which can hinder subsequent applications. Additionally, the intense heat and pressure applied may compromise the functional properties of the final product [49,50,51]. For example, bycatch fish have been utilized to produce nutrient-rich fish protein hydrolysates through acid hydrolysis using a combination of hydrochloric acid (4 M) and a papain-based enzyme at 100 °C for 90 min with 50.7% degree of hydrolysis [52]. In addition, Wang et al. [53] extracted fish skin gelatin hydrolysates using 6 M hydrochloric acid followed by enzymatic hydrolysis with Flavourzyme in the presence of 1% phenol at 110 °C for 24 h. On the other hand, alkali hydrolysis employs calcium, sodium, or potassium hydroxide as the alkaline agent to break down protein molecules. Unlike acid hydrolysis, this method works under milder conditions, requiring lower temperatures of around 54 °C. The significant drawbacks of alkali hydrolysis are the production of toxic by-products and the reduction in product functionality. Nevertheless, it remains a viable method for fish protein isolation to obtain bioactive protein hydrolysates [50,51,54]. Jack et al. [55] led alkali-based chemical hydrolysis of seaweed protein, achieving a 35.1% protein recovery. The process involved sonication (1 h at 42 Hz), salting out with 80% ammonium sulfate (*w*/*v*), and subsequent dialysis using a 3.5 kDa molecular weight cutoff (MWCO) membrane.

Bioactive peptides are predominantly extracted from marine-derived biomaterials through enzymatic hydrolysis, a generally recognized as safe (GRAS) method that enhances peptide stability and ensures experimental reproducibility. This technique is widely employed in the food, chemical, and pharmaceutical industries due to its eco-friendly nature, lower energy consumption, and minimal production of hazardous chemical waste. Enzymatic hydrolysis utilizes proteolytic enzymes (Table 1) derived from various biological sources, including plants (e.g., bromelain, pepsin), animals (e.g., trypsin), fungi (e.g., fungal proteases), and bacteria (e.g., alcalase, neutrase) [29,56]. These enzymes selectively cleave specific peptide bonds, releasing and contributing to the acidification of the reaction mixture. The process usually involves enzymatic inactivation at high temperatures, followed by centrifugation to separate the supernatant, and storage at −20 °C for subsequent use. The extraction efficiency of enzymatic hydrolysis is affected by critical parameters such as protease selection, enzyme concentration, pH, hydrolysis duration, and temperature. These factors significantly affect the resulting peptides’ yield, composition, and bioactivity. Therefore, optimizing hydrolysis conditions is essential to maximize peptide recovery and functional properties [6,57]. For instance, bulk fish protein hydrolysates exhibit varying angiotensin-converting enzyme (ACE) inhibitory capacity, as reported by Slizyte et al. [58], depending on the enzyme type (trypsin and Se-abzyme hydrolysates) and hydrolysis time (120 min). Similarly, Wang et al. [59] optimized the hydrolysis conditions for hairtail surimi peptides using response surface methodology combined with a Box–Behnken design. Their results indicated that an incubation time of 12.1 h, a temperature of 44.7 °C, and an enzyme concentration of 1858.8 U/g (dispase) resulted in peptides with enhanced antioxidant activity (29.7 ± 0.9 U/mL). Microbial fermentation is widely employed to produce marine peptides because of their GRAS status and several health benefits. This method involves microbial and enzymatic hydrolysis of protein substances to release bioactive peptides. The degree of hydrolysis is affected by several factors, such as the microbial strain, protein type, fermentation time, and temperature. For example, increased Zn concentrations in the culture of a fungus isolated from a hydrothermal vent crab (*Xenograpsus testudinatus*) led to the production of a novel anti-cancer cyclopeptide, clavatustide C [60]. Additionally, co-culturing different bacteria or yeast strains in a fermentation broth resulted in the production of distinct bioactive peptides compared with single-strain cultures. In a related study, Shin et al. [61] identified two novel piperazic acid-bearing cyclic peptides when Streptomyces sp. was co-cultured with *Bacillus* sp. Furthermore, fermentation broth conditions are crucial to optimizing marine peptide production. Bioactive peptides are produced during gastrointestinal digestion via sequential digestion by enzymatic (pepsin and pancreatin) proteolysis [62]. Under simulated in vitro digestion, Lin et al. [63] isolated novel antihypertensive peptides from *Rachycentron canadum* skin hydrolysates using sequential pepsin and pancreatin digestion. Similarly, Xia et al. [64] reported that sequential digestion of *Dunaliella salina* protein with pepsin and trypsin yielded a higher total peptide content (86.5%) compared with nondigested samples (46.8%). Furthermore, hydrolysate fraction from sea cucumber (*Stichopus japonicus*) protein was obtained through sequential digestion, first with simulated gastric fluid containing pepsin (2000 U/mL) at pH 3 for 2 h at 37 °C, followed by proteolysis with simulated intestinal fluid containing trypsin (100 U/mL) and chymotrypsin (25 U/mL) under the same conditions.

Marine-derived bioactive polypeptides obtained through protein degradation often contain impurities that necessitate further separation and purification to enhance their bioactivity and purity. Achieving high purity is crucial for accurately assessing their physicochemical and functional properties as well as ensuring safety and bioactive efficacy. The separation and purification of marine bioactive peptides involve various techniques, including chromatographic methods, membrane filtration, and capillary electrophoresis [65]. Membrane separation is typically the initial step in hydrolysate purification, allowing the fractionation of peptides based on molecular weight under applied pressure. Among membrane separation techniques, ultrafiltration is the most widely used due to its cost-effectiveness and environmental sustainability. For example, sea cucumber (*Stichopus japonicas*) peptides (SCPs), generated by α-chymotrypsin and fractionated into different molecular weights by ultrafiltration, exhibited varying antioxidant activities in H_2_O_2_-exposed Vero cells. The <5 kDa fraction showed the highest protection, reducing ROS generation and enhancing cell viability [66]. Chromatographic techniques, including ion-exchange chromatography, size-exclusion chromatography (gel filtration), affinity chromatography, and reverse-phase high-performance liquid chromatography (RP-HPLC), are commonly employed to purify bioactive peptide fractions further. Size-exclusion chromatography and RP-HPLC, which separate peptides based on size and hydrophobic interactions, are widely used for SCP purification. Jin et al. [39] purified antioxidant SCPs using a Sephadex G-25 gel column, an SP Sephadex C-25 ion-exchange column, and RP-HPLC, identifying a tetrapeptide (FLAP) with high antioxidant activity (EC_50_ = 0.385 mg/mL) through N-terminal amino acid sequencing. Capillary electrophoresis (CE) is an analytical method for peptide analysis based on m/z (mass-to-charge) values utilizing a fused silica capillary (typically, 100 cm × 100 μm). It offers advantages such as speed, low organic solvent consumption, cost-effectiveness, high efficiency, resolution, and selectivity. However, despite its high resolution and selectivity, CE is not widely used for purification due to its low injection volume and efficiency. Therefore, it is often considered a complementary technique to chromatography [67]. After a series of purification steps, marine peptides are identified using mass spectrometry (MS), bioinformatics analysis, and peptide sequencing techniques to determine their amino acid sequences. Mass spectrometry (MS) techniques, including matrix-assisted laser desorption/ionization time-of-flight (MALDI-TOF), liquid chromatography–mass spectrometry (LC–MS), electrospray ionization (ESI), and fast atom bombardment (FAB), are widely employed for peptide sequence confirmation due to their high efficiency and sensitivity. In addition, novel in silico approaches, such as molecular docking and quantitative structure–activity relationship (QSAR) models, are used for peptide production, overcoming the limitations of traditional purification and identification methods [68,69]. For example, novel angiotensin I-converting enzyme (ACE) inhibitory peptides were isolated from *Spirulina* protein hydrolysate using gel permeation chromatography based on molecular size. The peptide sequences were identified through LC-MS/MS followed by virtual screening and molecular docking to evaluate their ACE inhibitory potential [70].

**Table 1:** Common enzymes (proteases) used in the production of marine-derived peptides.

Enzyme (Protease)	References
Papain	[71,72,73]
Corolase	[74]
Thermolysin	[75]
Pancreatin	[76]
Ficin	[77]
Viscozyme	[78,79]
Trypsin	[73,80,81]
Pepsin	[64,82,83]
Chymotrypsin	[38,66]
Flavourzyme	[84,85]
Neutral protease	[86]
Alkaline protease	[14]
Subtilisin	[84]
Alcalase	[12,79,87,88,89]
Nucleicin	[90]
Orientase 22 BF	[90]
Neutrase	[12,39]
Protamex	[63,91,92]
Protease N	[63]
Corolase PP	[93]
Bromelain	[94]
Collagenase	[94]
Se-abzyme	[58]

## 4. Bioactivities of Marine-Derived Protein and Peptides

**Figure 3:**
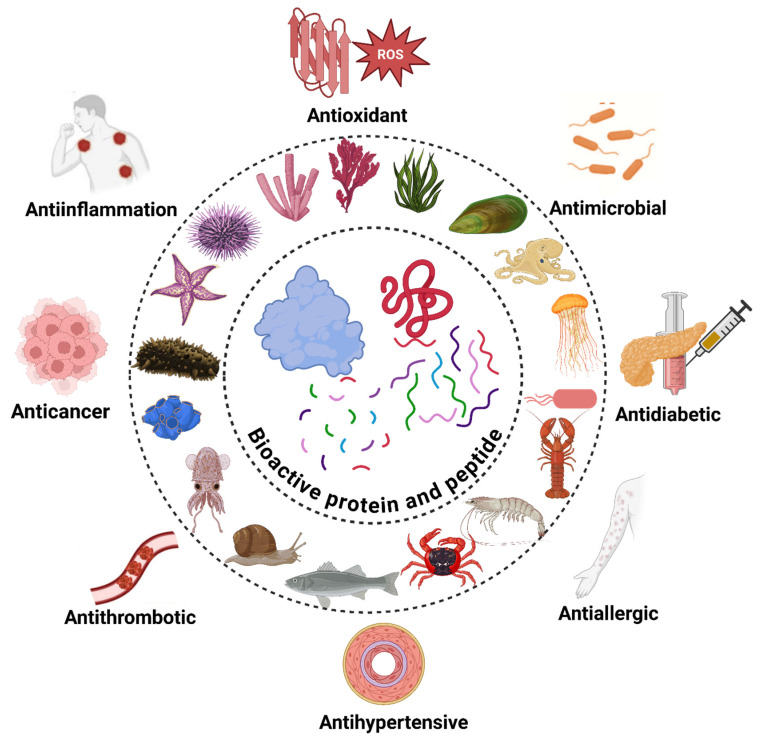
Bioactive potential of marine-derived proteins and peptides.

Marine organisms such as fish, algae, crustaceans, microbes, and invertebrates are leading sources of bioactive proteins and peptides, offering advantages like metabolic compatibility, the absence of religious restrictions, and freedom from terrestrial animal pathogens. These biomolecules exhibit diverse bioactivities (Table 2), including antioxidant, antihypertensive, antidiabetic, anticoagulant, antimicrobial, anti-inflammatory, antithrombotic, anti-cancer, and immunoregulatory effects. Figure 3 summarizes the bioactive potential of marine-derived proteins and peptides.

### 4.1. Antioxidant Activity

Oxidation is a necessary metabolism in the vertebrate and human body. However, it produces reactive oxygen species (ROS) and free radicals, which disrupt homeostasis and allow oxidative stress. Oxidative stress can be profoundly harmful to cells, potentially resulting in various diseases over time, including cardiovascular disease, stroke, arteriosclerosis, diabetes, and cancer [95,96,97,98,99]. Furthermore, the oxidation of lipids by ROS is of great concern to the food industry and consumers because it causes food deterioration and the production of toxic and off-flavor compounds. Lipid peroxidation is a problem in the food industry and human health [100,101,102]. Marine-derived proteins and peptides exhibit potent antioxidant properties ascribed to their capability to scavenge free radicals, chelate metal ions, and enhance endogenous antioxidant defense mechanisms. The antioxidant activity of these proteins and peptides has been evaluated through various in vitro assays, including scavenging of radicals such as those of 2, 2-diphenyl-1-picrylhydrazyl (DPPH), hydroxyl, superoxide anion, and ABTS, as well as metal ion chelation. The primary mechanism underlying their antioxidant potential implicates donating a hydrogen atom or an electron from the antioxidant molecules, effectively neutralizing free radicals and mitigating their harmful effects [103,104]. Hydrophobic amino acids, including phenylalanine (Phe, F), tryptophan (Trp, W), tyrosine (Tyr, Y), alanine (Ala, A), valine (Val, V), and leucine (Leu, L), contribute to the enhanced free radical scavenging activity of peptides by facilitating their interaction with and penetration into the lipid bilayers of target organ membranes through hydrophobic interactions. Meanwhile, essential and acidic amino acids, such as lysine (Lys, K), aspartic acid (Asp, D), and glutamic acid (Glu, E), act as efficient metal ion chelators. Additionally, aromatic amino acids like phenylalanine (Phe, F), tryptophan (Trp, W), and tyrosine (Tyr, Y) scavenge free radicals via direct electron transfer mechanisms [7]. 

Several studies have suggested that proteins and peptides from marine organisms possess potent antioxidant properties. Safari and Yaghoubzadeh [105] suggested that antioxidant peptides extracted from the sea cucumber (*Holothuria leucospilota*) could be used as a natural source of antioxidant compounds in the pharmaceutical and food industries. Protein hydrolysates were obtained from the body wall, processing by-product flower, and internal organs of the North Atlantic sea cucumber (*Cucumaria frondosa*) using the endopeptidases Alcalase (A), Corolase (C), and Flavourzyme (F). Among these, hydrolysates generated with (F + C) demonstrated the highest radical scavenging activity against DPPH and ABTS radicals, and the highest metal chelation activity was shown while using (C + F and A + F) [85]. In addition, protein hydrolysate isolated from *Stichopus japonicus* exhibited protective effects against oxidative stress. In H_2_O_2_-exposed Vero cells, treatment with the hydrolysate reduced the ROS levels, enhanced cell viability, and mitigated apoptotic damage. Similarly, in H_2_O_2_-exposed zebrafish embryos, the hydrolysate attenuated cell death and effectively decreased ROS and lipid peroxidation levels [66]. Hydrolysates derived from raw shrimp shell discards and shrimp shell protein, isolated using three other enzymes (trypsin, chymotrypsin, and pepsin), showed radical scavenging activities against ABTS, DPPH, and hydroxyl radicals as well as notable reducing power and ferrous ion chelating ability. These hydrolysates exhibited antioxidant activities in biological systems, including low-density lipoprotein (LDL) oxidation and DNA protection [106]. The antioxidant peptides isolated from Antarctic krill (*Euphausia superba*) showed strong reducing power, protective ability against H_2_O_2_-damaged plasmid DNA, and lipid peroxidation inhibition ability [107]. Guanghua et al. [108] reported that peptides derived from the pearl oyster (*Pinctada martensii*) mantle type V exhibited a more potent antioxidant activity than tilapia (*Oreochromis niloticus*) scale type I collagen, and *P. martensii* was a good source of natural antioxidants in the food-processing industry. Zhang et al. [109] reported that antioxidant peptides purified from *Mytilus coruscus* improved cell viability and ameliorated morphological damage in human umbilical vein endothelial cells (HUVECs). Novel bioactive peptides derived from fish hydrolysates, mainly from fish by-products, have been extensively explored. For example, Najafian and Babji [110] isolated three novel peptides from the myofibrillar protein hydrolysates of patin (*Pangasius sutchi*). Following purification and evaluation, these peptides demonstrated exceptional antioxidant activity, highlighting their potential as functional ingredients with antioxidative properties. Bashir et al. [111] found new antioxidant peptides in mackerel (*Scomber japonicus*) muscle protein hydrolysates. Among these, the peptide ALSTWTLQLGSTSFSASPM exhibited the highest DPPH scavenging activity. In contrast, the peptide LGTLLFIAIPI demonstrated the strongest superoxide dismutase (SOD)-like activity, further underscoring the potential of fish protein hydrolysates as sources of functional bioactive peptides. Zhang et al. [112] studied novel antioxidant peptides from gelatin skin hydrolysates of tilapia (*Oreochromis niloticus*). They found that the peptides Glu-Gly-Leu and Tyr-Gly-Asp-Glu-Tyr had potent hydroxyl radical scavenging activities. Alternatively, Saidi et al. [113] explored the valorization of tuna processing waste biomass. They identified four novel antioxidant peptides: YENGG, EGYPWN, YIVYPG, and WGDAGGYY. These peptides displayed vigorous scavenging activity against hydroxyl radicals. In addition, collagen peptides from the sea cucumber *Acaudina molpadioides*, especially Phe-Leu-Ala-Pro, exhibited strong antioxidant activity by scavenging DPPH radicals at 0.385 mg/mL. This effect was enhanced through Neutrase-assisted microwave hydrolysis, likely due to their small size and hydrophobic amino acids [39]. Kim et al. [114] found a new heptapeptide, TCGGQGR, from mackerel by-product hydrolysates. This peptide has strong antioxidant properties, making it useful for various applications. The protein hydrolysate of stripped weakfish by-product, obtained using the enzyme alcalase, demonstrated high DPPH radical scavenging activity, ranging from 60 to 70% [91]. The hydrolysis of jellyfish (*Rhopilema esculentum*) using Alcalase produced the peptides VKP and VKCFR, which enhanced free radical scavenging and protected rat cerebral microvascular endothelial cells (RCMECs) from H_2_O_2_-induced injury. They increased superoxide dismutase (SOD), catalase (CAT), and glutathione peroxidase (GSH-px) activities, highlighting their antioxidant potential [19].

Terriente-Palacios et al. [115] investigated the antioxidant capacities of enzymatic protein hydrolysates derived from various red (9 species), green (11 species), and brown (16 species) algae. Among the groups, red algae exhibited the highest median antioxidant activities, achieving 65.7 and 68.5% in DPPH radical scavenging and Trolox equivalent antioxidant capacity (TEAC) assays, respectively. This was followed by green algae (58.9 and 60.5%) and brown algae (47.5 and 49.7%), respectively. Notably, *Porphyra* sp., *Caulerpa lentillifera*, *A. platensis*, *C. vulgaris*, and *U. intestinalis* demonstrated antioxidant capacities exceeding 70% in either TEAC or DPPH assays. In contrast, most brown algae exhibited antioxidant capacities below 60%, except for *Odonella aurita*, *Nanochloropsis* sp., and *Bifurcaria bifurcate*.

While marine-derived proteins and peptides show well-documented antioxidant properties, they still present several gaps. While numerous studies highlight their radical scavenging and metal-chelating abilities, variations in extraction methods, structural characterization, and in vivo efficacy remain unresolved. Future research must standardize procedures, investigate structure–activity relationships, and conduct extensive in vivo experiments to validate their therapeutic application and feasibility.

### 4.2. Antimicrobial Activity

Studies have demonstrated that bioactive proteins and peptides possess significant antimicrobial activity. These bioactive compounds isolated from various sources, including marine organisms such as fish, invertebrates (i.e. Chelicerata, sea spiders, and crustaceans), amphibians, reptiles, and mammals, have been shown to exert inhibitory effects against diversified pathogenic microorganisms. Their antimicrobial activity is attributed to their ability to interact with the microbial cell membrane, rendering membrane disruption, inhibition of protein synthesis, or interference with essential cellular processes. Furthermore, bioactive peptides can promote the immune system, enhancing the host’s defense mechanisms. Due to their broad-spectrum activity and low potential for resistance development, bioactive proteins and peptides are considered promising candidates for developing novel medicinal agents and alternative treatments to conventional antibiotics [116,117]. Protein hydrolysates derived from marine sources exhibit a wide range of biological activities due to the various compounds and metabolites found in aquatic organisms. One such activity is antibacterial activity inhibiting bacterial growth [26]. For example, a study showed that protein extract from squid by-products, including viscera and ink sacs, demonstrated bioactivities capable of inhibiting the growth of bacteria and fungi [118]. White shrimp (*Litopenaeus vannamei*) carapaces show antibacterial activity after being conjugated with glucosamine. This modification improves the carapace’s bioactivity, making it effective at inhibiting bacterial growth [119]. Three novel antimicrobial peptides (Nv-p1, Nv-p2, and Nv-p3) were identified from the marine mollusk *Nerita versicolor*, demonstrating potent activity against pathogenic microorganisms. Notably, all three peptides exhibited antimicrobial activity against Pseudomonas aeruginosa, with Nv-p3 being the most active, displaying an inhibitory effect at a concentration as low as 1.5 µg/mL in radial diffusion assays [120]. EeCentrocin 1, a peptide isolated from sea urchins (*Echinus esculentus*), has demonstrated broad-spectrum antimicrobial activity against *Staphylococcus aureus*, *Escherichia coli*, *Staphylococcus epidermidis*, *Enterococcus faecalis*, *Micrococcus scarlatinae*, *Streptococcus pneumoniae*, *Pseudomonas aeruginosa*, *Klebsiella pneumoniae*, *Candida albicans*, and *Cryptococcus neoformans*. These peptides exert their antifungal effects by disrupting membrane integrity and reducing fungal adhesion, highlighting their potential as novel antimicrobial agents [121,122]. A synthetic 23-amino-acid peptide (GWLIRGAIHAGKAIHGLIHRRRH) derived from the defense protein three cDNA sequence of *Octopus minor* exhibited antifungal activity against *Candida albicans*, with a minimum inhibitory concentration (MIC) of 50 μg/mL and a minimum fungicidal concentration (MFC) of 200 μg/mL. The peptide-induced ultrastructural deformities in the *C. albicans* cell wall facilitated propidium iodide penetration, confirming the loss of membrane integrity and subsequent cell death at both MIC and MFC levels [123]. Similarly, a novel synthetic peptide, Octopromycin, derived from a prolin-rich protein from *Octopus minor*, exhibited antimicrobial activity against Acinetobacter baumannii, with a minimum inhibitory concentration (MIC) of 50 μg/mL and a minimum bactericidal concentration (MBC) of 200 μg/mL. The peptide altered the membrane permeability and induced a reactive oxygen species (ROS) burst, contributing to its bactericidal effect [124]. The antimicrobial Hepcidin-like (Lc-HepL) peptide, isolated from yellow croaker (*Larimichthys crocea*), showed antimicrobial activity against *Corynebacterium glutamicum, Micrococcus lysodeikticus,* and *Photobacterium damselae*. Its mechanism of action involves membrane damage and the stimulation of flocculant secretion, thereby limiting bacterial growth [125]. The antimicrobial peptide β-defensin, isolated from a marine fish, Ayu Plecoglossus altivelis, exhibited bactericidal activity against Vibrio anguillarum. Although the peptide showed limited antibacterial effects in vitro, it effectively boosted immune responses by enhancing phagocytosis, promoting intracellular bacterial killing, and stimulating the respiratory burst in monocytes/macrophages [126]. A novel antimicrobial peptide, polyphemusin III, was identified from the horseshoe crab *Limulus polyphemus* and evaluated against various bacterial strains. Polyphemusin III, with the amino acid sequence RRGCFRVCYRGFCFQRCR, exhibits structural homology to other β-hairpin peptides from the horseshoe crab. Its antimicrobial activity is attributed to disrupting plasma membrane integrity at a concentration IC_50_ of <10 μM, leading to cell death through a non-apoptotic mechanism. Its antimicrobial efficacy is comparable to or lower than other polyphemusins and tachyplesins [127]. Several studies have demonstrated that marine fish contain bioactive peptides with antimicrobial activity. For instance, three peptides—FEDQLR (HGM-Hp1), ALERTF (HGM-Hp2), and RHPEYAVSVLLR (HGM-Hp3)—were isolated from Half-Fin Anchovy (*Setipinna taty*) hydrolysates and exhibited antimicrobial activity. HGM-Hp3 significantly enhanced intracellular H_2_O_2_ production in *E. coli*, while HGM-Hp1 and HGM-Hp2 induced potassium ion leakage, indicating irreversible membrane damage and cell integrity disruption [128]. Additionally, a series of short peptides with antimicrobial activity were derived from Barbel muscle protein hydrolysates and *Sardinella aurita* hydrolysates, highlighting their potential as bioactive compounds [129,130].

Although the antimicrobial activity of proteins and peptides from marine sources is crucial for further applications, there are key knowledge gaps, including peptide stability, bioavailability, unclear mechanisms of action, potential resistance development, and limited in vivo studies. Addressing these structural modifications and nanoparticle delivery can enhance stability, while omics studies can clarify mechanisms. Long-term resistance studies and combination therapies with antibiotics can help mitigate resistance risks. More in vivo and clinical trials are needed to confirm therapeutic potential, and recombinant expression systems can improve large-scale production efficiency. Addressing these gaps will strengthen the clinical relevance of marine-derived peptides.

### 4.3. Anti-Inflammatory Effects

Inflammation is the body’s first immune response to toxins, pathogens, allergens, and injury, involving complex interactions between soluble factors and immune cells [131]. It recruits leukocytes and activates the release of IL-1, IL-6, TNF-α, PGE2, and key enzymes like COX-2 and iNOS, facilitating recovery. Pathogen distinction receptors such as Toll-like receptors (TLRs) detect pathogens to initiate immune responses. Anti-inflammatory treatments include non-steroidal anti-inflammatory drugs (NSAIDs) and steroids, though NSAIDs can cause adverse effects like ulcers and, rarely, cardiovascular events [103,132]. Multiple studies have shown that marine organisms serve as a rich reservoir of bioactive proteins, peptides, and amino acids with potent anti-inflammatory properties. These naturally derived compounds regulate key inflammatory pathways. For instance, the marine crab (*Charybdis natator*) leg muscle-derived peptide LGLGAAVL (713.45 Da) exhibits anti-inflammatory activity by suppressing LPS-induced COX-2 expression in macrophage cells, highlighting its potential as a natural therapeutic agent [133]. The pearl oyster (*Pinctada martensii*) meat hydrolysate-derived peptides TWP (402.19 Da), TAMY (484.19 Da), and FPGA (390.19 Da) exhibit anti-inflammatory effects. These peptides are attributed to reducing NO and pro-inflammatory cytokines (TNF-α, IL-6, IL-1β) while enhancing IL-10 production, making them promising candidates for inflammation management [134]. A novel peptide, EGLLGDVF (849.43 Da), isolated from the green mussel (*Perna viridis*) foot, has demonstrated anti-inflammatory properties. This peptide has been found to reduce the production of pro-inflammatory cytokines, suppress nitric oxide (NO) levels, and inhibit COX-2 activation [87]. The ventral harp (*Harpa ventricosa*) visceral mass-derived peptide ALGTWK (690.20 Da) demonstrates remarkable stability and potent anti-inflammatory activity in LPS-stimulated RAW264.7 macrophages. This bioactive peptide modulates inflammatory signaling pathways by downregulating NO, TNF-α, and IL-1β production [135]. Hydrolysates derived from the sea cucumber (*Apostichopus japonicus* and *Acaudina leucoprocta*), containing the bioactive peptides GPSGRP (569.60 Da), GPAGPR (553.60 Da), PQGETGA (658.64 Da), and GFDGPEGPR (930.95 Da), show anti-inflammatory properties. These peptides effectively lessen the expression of TNF-α, IL-1β, and IL-6 by inactivating the MAPK/NF-κB signaling pathway in an LPS-induced liver injury mouse model [136]. Two bioactive peptides isolated from an aqueous extract from Baijiao sea bass (*Lateolabrax maculatus*) demonstrated anti-inflammatory activity by inhibiting and suppressing nitric oxide (NO) production in LPS-induced macrophages [137]. The anti-inflammatory peptide (LLFTTQ, 721.80 Da) was isolated from powder extract from skipjack tuna (*Katsuwonus pelamis*). The peptide has been found to reduce IL-6, IL-10, and TNF-α levels, key cytokines involved in intestinal inflammation [128]. Salmon (*Salmo slalar*) containing a bioactive peptide (PAY, 349.37 Da) extracted from the pectoral fin hydrolysate showed anti-inflammatory activity by preventing the NO/iNOS and PGE2/COX-2 pathways. Additionally, it reduced the production of pro-inflammatory cytokines such as TNF-α, IL-6, and IL-1β [138]. A new peptide isolated from the muscle of the sturgeon (*Acipenser schrenckii*) demonstrates anti-inflammatory effects by suppressing MAPK phosphorylation and inhibiting the MAPK pathway [139]. Marine bacteria-derived peptides have demonstrated anti-inflammatory activity. Specifically, peptides from *Synechococcus* sp. trypsin hydrolysate—AILQSYSAGKTK, ALNKTHLIQTK, LLVHAPVK, IPDAHPVK, and VVVLRDGAVQQLGTPR—with molecular weights of 1265.69, 1265.74, 876.11, 875.48, and 1706.97 Da, respectively, have been shown to suppress the gene expression of pro-inflammatory cytokines, including iNOS, TNF-α, COX-2, and IL-6 [80]. The microalga *Porphyridium* sp. hydrolysate contained peptides such as GVDYVRFF, AIPAAPAAPAGPKLY, and LIHADPPGVGL, with molecular weights of 1002.11, 1407.65, and 1088.25 Da, respectively, which have been reported to inhibit COX-1 by 92.14% and COX-2 by 32.25% compared with the resveratrol control [78]. Research has shown that marine-derived mollusks possess anti-inflammatory properties due to their diverse peptide content. For instance, a peptide identified in the foot of the Asiatic hard clam (*Meretrix meretrix*), NPAQDC, with a molecular weight of 647.55 Da, has been reported to constrain pro-inflammatory cytokine expression and nitric oxide (NO) production while suppressing COX-2 activation in LPS-stimulated macrophage cells [87]. Marine worms have been recognized for their anti-inflammatory properties due to the presence of bioactive peptides. For example, a hydrolysate from the clam worm (*Marphysa sanguinea*) containing the peptide NCWPFQGVPLGFQAPP (1757.86 Da) has been shown to suppress the excessive production of pro-inflammatory cytokines, including NO, iNOS, TNF-α, and COX-2, in LPS-stimulated RAW264.7 macrophages. Similarly, the peanut worm (*Sipunculus nudus*) alcalase hydrolysate, which includes the peptides TVNLAYY (843.42 Da) and LSPLLAAH (821.48 Da), has been demonstrated to inhibit NO production in RAW 264.7 macrophages without cytotoxic effects while also downregulating the expression of key pro-inflammatory cytokine genes, such as iNOS, IL-6, TNF-α, and COX-2 [140,141]. Marine-derived protein hydrolysates have been recognized for their anti-inflammatory properties. Notably, a hydrolysate with low molecular weight fraction (<10 kDa) obtained from sardine (*Sardina pilchardus*) by-products from the canning industry was shown to have significant anti-inflammatory effects [142].

Marine-derived peptides exhibit anti-inflammatory activity through several key mechanisms. One of the most common pathways involves inhibiting NF-κB, a transcription factor that controls the expression of pro-inflammatory cytokines, including TNF-α, IL-6, and IL-1β. Additionally, many peptides stop the MAPK signaling pathway, including ERK, JNK, and p38, decreasing inflammation-related cellular responses. Another widely accepted mechanism is the downregulation of NO production and iNOS expression, which mitigates oxidative stress and inflammatory damage. Furthermore, marine peptides often hinder the COX-2/PGE2 pathway, leading to a decrease in prostaglandin-mediated inflammation. Many of these bioactive compounds also exhibit immunomodulatory effects by reducing pro-inflammatory cytokines (TNF-α, IL-6, IL-1β) while enhancing the production of anti-inflammatory cytokines like IL-10. These mechanisms collectively contribute to the potent anti-inflammatory effects of marine-derived proteins and peptides, making them promising candidates for therapeutic applications in inflammation-related diseases.

Although the impressive anti-inflammatory activity of peptides from marine origins has been well documented, knowledge gaps still exist in covering their specific molecular interactions, bioavailability, and clinical activity. While many studies suggest their ability to modulate inflammatory pathways such as NF-κB, MAPK, and COX-2/PGE2, inconsistency in peptide extraction, structural characterization, and in vivo validation limits their translational potential. Future research should be focused on optimizing extraction and purification procedures, clarifying structure–activity relationships, and conducting intricate in vivo and clinical trials to establish their therapeutic applications against inflammation-related disorders.

### 4.4. Antihypertensive Effects

Hypertension is a severe chronic disease and a major global health concern among adults. Despite the availability of various angiotensin I-converting enzyme (ACE) inhibitor drugs, the mortality rate associated with hypertension continues to rise. Angiotensin-converting enzymes play a critical physiological role in regulating blood pressure. They catalyze the hydrolysis of angiotensin I, an inactive decapeptide, into angiotensin II, a potent vasoconstrictor inhibiting bradykinin’s vasodilatory effects, resulting in a rise in blood pressure. Consequently, the inhibition of ACE activity is a key therapeutic strategy for reducing the risk of hypertension and managing high blood pressure [143]. Numerous studies have highlighted the potential of proteins and bioactive peptides derived from aquatic resources as natural ACE inhibitors. For example, peptides like IK, YEGDP, WF, and SWISS, derived from blue mussels (*Mytilus edulis*) through enzymatic hydrolysis, exhibited the most muscular ACE inhibitory activity (with IC_50_ values of 0.77 ± 0.020, 0.19 ± 0.010, 0.40 ± 0.015, and 0.32 ± 0.017 mg mL^−1^, respectively). This inhibitory effect is attributed to their efficient interaction with the ACE active site, facilitated by hydrogen bonding, electrostatic forces, and hydrophobic interactions [70].

Furthermore, two peptides, VKP and VKCFR, isolated from the jellyfish *Rhopilema esculentum*, exhibit significant inhibition of angiotensin-I-converting enzyme (ACE) with IC_50_ values of 1.3 μM and 34.5 μM, respectively. As demonstrated by molecular docking studies, these peptides bind to the ACE active site, specifically coordinating with the Zn (II) ion [115]. Peptides extracted from monkfish (*Lophius litulon*) swim bladders through acid and enzymatic hydrolysis demonstrated significant ACE inhibitory activity. Similarly, bioactive peptides derived from shortfin scad (*Decapterus macrosoma*) waste via alcalase hydrolysis exhibited potent ACE inhibitory properties [12]. Further, peptides extracted from marine algae have also demonstrated ACE inhibitory activity. For instance, the peptide FQIN[M(O)]CILR, derived from the red alga *Gracilariopsis lemaneiformis* (Rhodophyta), exhibited significant ACE inhibitory effects. This peptide effectively reduced blood pressure in spontaneously hypertensive rats [144]. Additionally, the peptide PIZ from microalgae *Isochrysis zhanjiangensis* inhibits ACE in a non-competitive manner and mitigates Ang II-induced vascular inflammation and apoptosis by modulating the NF-κB, Nrf2, MAPKs, and Akt pathways [145]. The peptide sequence Thr-Tyr-Ile-Ala, derived from the marine alga *Porphyra dioica* through enzymatic hydrolysis, demonstrated the highest ACE inhibitory activity [146]. Wang et al. [102] successfully isolated eight antihypertensive peptides (FY, KY, AKY, GKY, KFKY, KKFY, SKTY, and AKYSY) from *Laminaria japonica*, all of which feature tyrosine (Tyr) residues at their C-terminal positions. These findings underscore the significant role of Tyr residues in enhancing the antihypertensive bioactivity of the peptides. Three dipeptides, namely Ile-Trp, Leu-Trp, and Val-Trp, isolated from *Chlorella sorokiniana*, demonstrated remarkable inhibition against angiotensin-converting enzyme (ACE). This enhanced activity is likely attributed to aromatic tryptophan (Trp) residues at the carboxy termini coupled with branched-chain hydrophobic amino acids at the nitrogen termini of these peptides [147]. An angiotensin-converting enzyme (ACE) inhibitory peptide, ETT, was identified from *Isochrysis zhanjiangensis* and demonstrated significant bioactivity. ETT effectively inhibits ACE through a non-competitive binding mechanism, with an IC_50_ of 15.08 μM. Furthermore, it suppresses angiotensin II-induced inflammation and apoptosis, suggesting potential cardioprotective properties. In vivo studies in spontaneously hypertensive rats further confirmed its antihypertensive effects [81]. Another peptide, PIZ, isolated from *Isochrysis zhanjiangensis*, exhibits ACE inhibition with an IC_50_ of 61.38 μM through a non-competitive binding mode. Additionally, PIZ suppresses angiotensin (Ang II)-induced vascular factor secretion [145]. Anekthanakul et al. [5] used in silico and in vitro approaches to identify a potent ACE-I inhibitory peptide sequenced as IRDLDYY from spirulina (*Arthrospira platensis*). This peptide exhibited a remarkable IC_50_ value of 1.75 mM, reflecting its efficacy in halving enzyme activity. It was demonstrated to be non-toxic to human cells.

Shrimp shell waste has also been reported to exhibit ACE inhibitory properties. Using different proteases, Feng et al. [143] isolated ACE inhibitory peptides from shrimp shell waste. Among the hydrolysates, those produced by neutral protease, containing peptides with molecular weights < 5 kDa, exhibited the highest ACE inhibitory activity, achieving 84.04%. The sea cucumber has been identified as a natural source of ACE inhibitory activity. For example, *Actinopyga lecanora* proteolysate (ALP) has demonstrated significant ACE inhibition in vivo, exhibiting both preventive and therapeutic effects in hypertensive Sprague-Dawley rats [148]. Similarly, bioactive peptides isolated from *Stichopus horrens* have demonstrated ACE inhibitory properties. Specifically, the peptides EVSQGRP, CRQNTLGHNTQTSIAQ, and VSRHFASYAN showed ACE inhibitory activity with IC_50_ values of 0.05 mM, 0.08 mM, and 0.21 mM, respectively, as determined by the HHL method [149].

Therefore, bioactive peptides from marine sources can regulate hypertension by inhibiting ACE. While their antihypertensive effects have been revealed in vivo employing animal models, further human intervention studies are needed to verify their health benefits.

### 4.5. Anti-Cancer Potential

Cancer is a group of diseases caused by uncontrolled cell division and the spread of abnormal cells. It happens when DNA mutations disrupt the normal processes that control cell growth and death, leading to tumor formation. Cancer can change cells and genes and spread to other body parts [150]. Anti-carcinogenic, anti-cancer, and anti-proliferative activities refer to the ability to counteract carcinogens’ effects or inhibit cancer cell development. Anti-carcinogens or anti-cancer agents selectively target and either destroy or suppress the growth of cancer cells. Bioactive peptides with anti-cancer activity offer promising therapeutic strategies by selectively targeting cancer cells with minimal toxicity to healthy tissues, potentially reducing side effects. Plants, animals, microbes, and marine species are significant sources of bioactive peptides [151].

Marine-derived bioactive peptides exhibit anti-cancer activity through multiple mechanisms, including tumor suppression and the induction of apoptosis in malignant cells. A key mechanism is the activation of apoptosis via the intrinsic mitochondrial pathway, where peptides trigger the release of cytochrome c from mitochondria, initiating a caspase cascade that results in programmed cell death. Specific peptides also regulate apoptosis-related proteins by upregulating pro-apoptotic proteins like Bax and downregulating anti-apoptotic proteins like Bcl-2, further promoting cancer cell death. Additionally, these peptides inhibit angiogenesis by targeting vascular endothelial growth factor (VEGF), depriving tumors of essential nutrients and oxygen. By modulating overactive signaling pathways, such as PI3K/Akt and MAPK, marine peptides suppress tumor growth and enhance the efficacy of existing cancer therapies [152,153,154].

Bioactive peptides, mainly those derived from fish hydrolysates, can reduce oxidative stress by lowering reactive oxygen species (ROS), thereby preventing genetic alterations like mutations and chromosomal aberrations, which play key roles in carcinogenesis. A study on the anti-proliferative effects of protein hydrolysates from fish by-products tested on human colon and breast cancer cells showed that fish protein hydrolysates (FPHs) from the skin, bones, head, and viscera of various species effectively inhibited cancer cell growth [33]. Additionally, Hamzeh et al. [155] investigated the anti-proliferative and antioxidative activities of cuttlefish (*Sepia pharaonic*) protein hydrolysates. The study revealed that the fish protein hydrolysates (FPHs) significantly inhibited the growth of MDA-231 and T47D cancer cells with growth inhibition rates of 78.2 and 66.2%, respectively. In addition to protein hydrolysates, fish-derived anti-cancer peptides (ACPs), primarily found in mucus, include pardaxin, piscidin, and epinecidin-1. Pardaxin, from the Red Sea Moses sole (*Pardachirus marmoratus*), is the most studied, with a 33 amino acid α-helical structure. It exhibits anti-cancer activity by inducing G2/M phase cell cycle arrest, thereby inhibiting cancer cell proliferation [156]. In addition, pardaxin has been investigated for its antitumor properties, demonstrating the ability to induce apoptotic cell death in various cancer cell lines, including oral squamous carcinoma, ovarian cancer, and cervical carcinoma [137,157]. The piscidin family exhibits extensive anti-cancer activity. For instance, piscidin-4 has shown significant cytotoxicity against non-small-cell lung cancer (NSCLC) cell lines, including A549, NCI-H661, NCI-H1975, and HCC827 [157]. Notably, piscidin-4 induced NSCLC cell death through the necrotic rather than the apoptotic pathway. Epinecidin-1, a naturally occurring peptide isolated from the orange-spotted grouper (*Epinephelus coioides*), exhibits potent anti-cancer activity. It has been shown to inhibit human lung cancer and glioblastoma by regulating apoptosis and necrosis [32,158].

Yu et al. [159] investigated *Cyclina sinensis* protein hydrolysates (CSPs) to develop novel pentapeptides with anti-proliferative properties capable of inducing apoptosis in prostate cancer cells. The study demonstrated that the hydrolysates derived from *C. sinensis* significantly inhibited the development of DU-145 prostate cancer cells. As filter-feeding organisms, sponges produce neutralizing bioactive compounds, including peptides, to manage harmful environmental particles. Over the past two decades, research has focused on sponge-derived peptides such as jaspamide, koshikamides, and theonellamide G, demonstrating significant cytotoxicity against various cancer cell lines [160]. These peptides are distinguished by unique amino acid or non-amino-acid components compared with other animal-derived anti-cancer peptides (ACPs). Jaspamide and koshikamides are uniquely structured cyclic depsipeptides identified in the sponges *Jaspis johnstoni* and *Theonella* species, respectively. These compounds have shown cytotoxic activity against various cancer cells, including prostate and breast carcinomas, acute myeloid leukemia, and colon cancer cells [34,161]. Theonellamide G is a glycopeptide derived from *Theonella swinhoei*, showing cytotoxic effects against the HCT-16 human colon adenocarcinoma cell line [9]. The anti-cancer properties of marine ascidians have been widely reported. For example, turgencin A and turgencin B, two novel linear antimicrobial peptides (ACPs) containing six cysteine residues, were isolated from the ascidian *Synoicum turgens*. These peptides exhibited significant anti-cancer activity by suppressing the proliferation of the melanoma cancer cell line A2058 and the human fibroblast cell line MRC-5 [162].

The anti-cancer properties of sea cucumbers have been well documented. For example, sea cucumber hydrolysate has been shown to inhibit the proliferation, migration, and invasion of A549 lung cancer cells. Additionally, it suppresses pleural effusion formation, reduces lung tumor growth, and extends survival time in cancer-bearing C57BL/6 mice [163]. In addition, peptides (WPPNYQW and YDWRF) from *Cucumaria frondosa* were docked with four proteins, EGFR, PI3K, AKT1, and CDK4, revealing strong binding interactions. Molecular dynamics simulations showed that WPPNYQW formed stable complexes with all four proteins, while YDWRF was stable only with PI3K and AKT1. Due to its broad and stable interactions, WPPNYQW exhibits high potential as an anti-breast-cancer agent [164].

Although peptides derived from the marine environment show promising anti-cancer potential, several gaps in knowledge need to be addressed, including comprehensive in vivo studies, precise mechanisms of action, and correlation with the clinic. Many peptides show activity in vitro, but their bioavailability, stability, and pharmacokinetics in humans must be more thoroughly examined. Large-scale synthesis, purification, and cost-effectiveness for therapeutic applications are also complicated. Advanced drug delivery systems such as nanocarriers must overcome these hurdles and improve peptide stability and targeted delivery. Further, integrating computational approaches such as molecular docking and AI-driven screening can expedite peptide selection for clinical trials. Interdisciplinary collaborations between marine biotechnologists, pharmacologists, and clinicians will accelerate the translation of these bioactive compounds from the lab to the clinic.

### 4.6. Immunomodulatory Effects

The human immune system is crucial in preventing and controlling infections and neoplasia through cellular and humoral mechanisms. However, various factors can compromise immune function, including malnutrition, psychological and oxidative stress, and exposure to exogenous pathogens and antigens. While pharmaceuticals such as actinomycin, vincristine, dexamethasone, levamisole, and Thymosin α1 serve as immunomodulators, their high cost and potential side effects limit their suitability for long-term use. Therefore, nutrition-based interventions, such as exceptionally functional foods containing immunomodulatory peptides, have gained attention for their role in immune regulation. While immunomodulatory peptides are known to boost immune functions like lymphocyte proliferation, natural killer (NK) cell activity, and cytokine regulation, their precise mechanisms of action remain uncertain [26,86,165].

Marine organisms, as a rich source of bioactive proteins and peptides, offer natural immunoregulatory properties. These bioactive compounds and protein hydrolysates hold promise as effective and sustainable alternatives for enhancing immune and immunoregulatory function and overall health. For example, a protein hydrolysate from the skin of the giant croaker (*Nibea japonica*) has been shown to enhance the immune system by promoting both cell-mediated immunity, such as increased splenocyte proliferation, and humoral immunity, including elevated immunoglobulin levels [86]. An immunomodulatory peptide was isolated from *Stolephorus chinensis*, with its amino acid sequence identified as Tyr-Val-Met-Arg-Phe. The peptide fraction, with a molecular weight of <1 kDa, exhibited the highest relative proliferation rate (RPR) in RAW 264.7 cells (70.03%) [14]. The immunoregulatory properties of sea cucumbers have been reported, particularly in hydrolysates obtained from enzymatic hydrolysis of *Apostichopus japonicus*. These hydrolysates enhance the immune response by upregulating both mRNA levels and the secretion of NO, TNF-α, and IL-6 in a dose-dependent manner. Additionally, they stimulate macrophages by modulating the NF-κB and MAPK signaling pathways in RAW264.7 cells [166]. Similarly, oligopeptides from the enzymatic hydrolysis of sea cucumber (*Codonopsis pilosula*) have been shown to enhance immune function by boosting cell-mediated and humoral immunity, promoting macrophage phagocytosis, and increasing NK cell activity in BALB/c mice [167]. Furthermore, a hydrolysate fraction from the sea cucumber (*Colochirus robustus*), obtained through enzymatic hydrolysis, has been found to enhance immune function by stimulating lymphocyte proliferation, increasing serum albumin levels, and boosting natural killer (NK) cell activity. Additionally, it stimulates helper T-cell functions in C57BL/6 mice [168]. However, in regulating inflammation, proteins and peptides have the potential as drug candidates for modulating innate immunity. Developing protein- and peptide-based drugs may help mitigate adverse immune reactions associated with traditional drugs while enhancing immune efficacy [26].

### 4.7. Antihyperlipidemic Properties

Obesity is an excessive or abnormal fat accumulation that results from excessive dietary intake, particularly a high-fat diet (HFD), which is a well-established risk factor for hyperlipidemia, fatty liver disease, hypertension, and type 2 diabetes mellitus [169,170]. Hyperlipidemia is a substantial risk factor for cardiovascular diseases (CVDs), as it increases the likelihood of their development. It commonly manifests as hypercholesterolemia and hypertriglyceridemia, both of which are prevalent among CVD patients [6]. Bioactive peptides isolated from fish protein hydrolysates and algal glucans have been shown to possess hypocholesteric effects. For example, bioactive peptides extracted from microalgal glucans (polysaccharides) strengthen the immune system and exhibit potent antioxidant and cholesterol-lowering effects [171]. Wang et al. [117] identified novel hypocholesterolemic peptides (TKY, LIL, FPK, and IAIM) from silver carp muscle that function through two mechanisms: inhibiting cholesterol absorption and promoting the uptake of peripheral low-density lipoprotein (LDL). Moreover, fish protein hydrolysates from sardine, horse mackerel, axillary seabream, bogue, small-spotted catshark, and blue whiting have been recognized as effective for formulating cholesterol-lowering supplements. Furthermore, the peptides LLRLTDL and GYALPCDCL, isolated from Ark shell protein hydrolysate, demonstrate significant anti-obesity effects by inhibiting lipid accumulation. At a concentration of 100 μM, lipid accumulation was reduced by 48.53 and 46.22%, respectively, through a combination of antioxidant and anti-inflammatory effects and by directly modulating adipocyte differentiation [169]. Fish collagen peptides (FCPs) are derived from the skin of great hammerhead sharks (*Sphyrna mokarran*) to attenuate high-fat diet and alcohol-induced hyperlipidemia. Results revealed that the peptide modulates lipid metabolism by downregulating fatty acid synthase (FAS) and 3-hydroxy-3-methylglutaryl-coenzyme A reductase (HMGCR) while upregulating lecithin-cholesterol acyltransferase (LCAT) expression in the liver, thereby reducing cholesterol accumulation [101]. Similarly, collagen peptides derived from Raja kenojei skin have demonstrated potential in modulating lipid metabolism and exerting anti-obesity effects in mice fed a high-fat diet. These peptides inhibit lipogenesis, suppress adipocyte differentiation, and enhance fatty acid oxidation. Notably, adipose tissue size was reduced from 3% in the control group to 1.5% in the peptide-treated group [172,173]. Additionally, Lee et al. [174] found that enzymatically derived tuna skin collagen peptides inhibited lipid accumulation in 3T3-L1 cells and reduced serum TC, TAG, and LDL levels while increasing serum high-density lipoprotein (HDL) levels in HFD-induced obese mice. The hypolipidemic activity of two peptides, VIAPW and IRWWW, isolated from *Miichthys miiuy* muscle digests, was demonstrated by their significant, dose-dependent inhibition of oleic acid (OA)-induced lipid accumulation in HepG2 cells along with a reduction in intracellular triacylglycerol (TAG) and total cholesterol (TC) levels. Both peptides downregulated lipid synthesis-related genes (SREBP-1c, SREBP-2, FAS, ACC, and HMGCR) and upregulated genes involved in lipid oxidation (PPARα, ACOX-1, and CPT-1) [59]. Studies have shown that dietary protein hydrolysates from marine fish affect blood cholesterol concentrations and antioxidant status. For instance, protein hydrolysate from *Boops boops* reduced serum levels of TC, TAG, LDL, HDL, ALT1, ALP2, and AST3; decreased liver MDA levels; and enhanced liver antioxidant enzyme activity in rats fed a high-cholesterol diet [175]. Furthermore, sardine and sardinelle protein hydrolysates show potent cholesterol-lowering effects, reducing lipid peroxidation in serum and target tissues while enhancing antioxidant enzyme activity in rats fed a high-cholesterol diet [176]. Therefore, these findings suggest that marine-derived proteins and peptides hold promise for potential applications in developing anti-obesity drugs.

Despite encouraging outcomes on the hypolipidemic and anti-obesity effects of marine-derived proteins and peptides, several knowledge gaps remain. Firstly, the precise molecular mechanisms of their lipid-lowering and anti-adipogenic impact need to be explored, mainly how they affect the pathways of lipid metabolism. Secondly, most studies are limited to in vitro and animal models, with well-planned clinical trials necessary to confirm their efficacy and safety in humans. Furthermore, these peptides’ bioavailability, stability, and optimal dosage in foods or pharmaceuticals are unknown. The future must include the identification of peptide sequences with higher potency, optimizing delivery systems for more excellent stability and uptake, and conducting large-scale human studies to establish their therapeutic utility for obesity and related metabolic diseases.

### 4.8. Wound Healing and Skin Health

The skin epidermis is a crucial innate defense barrier against pathogens that plays a key role in tissue homeostasis. Skin injuries arising from burns, infections, scarring, genetic disorders, and other diseases are challenging to treat and increasingly common. Treatments focus on restoring tissue integrity through inflammation, cell division, differentiation, and vascularization. Endothelial permeability permits cell adhesion, directing to cell differentiation and maturation. Marine collagen has been demonstrated to be an effective biomaterial for wound healing. It can be utilized in mixed forms, including collagen peptides, hydroxylates, collagen fibers, and scaffold-like structures [177,178]. Hu et al. [177] demonstrated through an in vitro scratch assay that marine collagen peptides improve wound closure at 50 μg/mL concentrations starting 12 h post-treatment. At this concentration, cell migration was comparable to that induced by 10.0 ng/mL of epidermal growth factor, a key factor in wound healing. Furthermore, rabbits with wounds treated with marine collagen peptides derived from tilapia skin showed significantly faster healing after 11 days than the control group. Yang et al. [179] isolated collagen peptides from Alaska pollock and showed that oral administration to wounded rats significantly improved recovery rates compared with control groups. Hydroxyproline, which promotes collagen deposition and healing, was higher in the collagen-treated group (10.6 µg/mg) over time than the control group (9.25 µg/mg). On day 12 of healing, the treated groups showed complete re-epithelialization and the presence of hair follicles, while the control group showed poor keratinocyte migration and no hair follicles. Wang et al. [53] found that marine collagen peptides (MCPs) derived from salmon skin significantly enhanced skin wound tensile strength in rats. Additionally, Pozzolini et al. [180] isolated and purified marine collagen hydroxylates from the marine sponge *Chondrosia reniformis*. In an in vitro scratch assay, collagen peptide fractions at 50 μg/mL were added, and cells were analyzed at 0, 6, 24, and 30 h post-treatment. Compared with control groups, the treated cells showed fibroblast and keratinocyte migration and proliferation, improving wound gap closure in dermal and epidermal cells. Migration and colonization of the scratch gap were observed initially, followed by increased cell proliferation at the 24-hour mark. These findings underscore the promising wound-healing potential of marine collagen hydroxylates from *C. reniformis*. Furthermore, Dang et al. [181] investigated the biological activity of acid-soluble collagen (ASC) hydrolysates derived from haddock (*Melanogrammus aeglefinus*) skin in wound healing. The study demonstrated that ASC enhances wound healing through several mechanisms, including promoting vascularization, stimulating epithelial cell regeneration, and accelerating wound closure. Additionally, ASC was found to reduce bleeding and clotting times, further contributing to its wound-healing efficacy. Veeruraj et al. [82] isolated astaxanthin and acid- and pepsin-soluble collagen from the squid (*Doryteuthis singhalensis*). In their study, wounded rats treated with a combination of astaxanthin and collagen demonstrated a faster wound healing rate than those treated with saline. The collagen-treated groups showed enhanced epithelization, angiogenesis, keratinization, and the presence of collagen fibers, all of which contributed to the accelerated wound-healing process. Similarly, collagen isolated from marine tilapia skin and bovine skin collagen nanofibers demonstrated that collagen-treated rat groups exhibited faster wound recovery rates than controls. The investigation also emphasized the significant role of hydroxyproline, a pivotal component of collagen, in accelerating wound healing by promoting re-epithelization. The collagen-treated groups showed increased fibroblast activity, enhanced vascularization, reduced inflammation, and a higher presence of collagen fibers than the control groups [182]. Moreover, Melotti et al. [183] developed collagen-based skin-like scaffolds (CBSSs) from sea urchin food waste to treat skin wounds in sheep. Their study revealed that CBSS-treated wounds healed faster than the controls, with significantly more keratinocyte migration by day 14. The treated groups also had reduced inflammation and more significant deposition of granular tissue compared with the controls. These results highlight the superior properties of marine-derived protein, mainly collagen, in skin regeneration, suggesting its potential applications in the pharmaceutical and cosmeceutical industries.

### 4.9. Antidiabetic Properties

Diabetes mellitus (DM) is a significant public health concern, projected to affect 700 million people by 2045, with type 2 diabetes (T2D) accounting for approximately 90% of cases. T2D is a metabolic disease linked to insulin resistance and influenced by diet and lifestyle. Due to the side effects of existing remedies, dietary proteins and peptides from natural sources are gaining interest as potential therapeutic agents [7,184]. Dipeptidyl peptidase IV (DPP-IV) is a key therapeutic target for T2D, as it deactivates the incretin hormones glucagon-like peptide 1 (GLP-1) and glucose-dependent insulinotropic polypeptide (GIP), which regulate postprandial insulin secretion and blood glucose levels. Additionally, inhibiting carbohydrate-digesting enzymes, such as α-amylase and α-glucosidase, can reduce intestinal glucose absorption [7]. Numerous studies have shown that marine-derived proteins and peptides exhibit antidiabetic activity by inhibiting enzymes involved in carbohydrate digestion. Bioactive peptides from various algae species have shown antidiabetic properties, with most studies focusing on *A. platensis*, *Chlorella* sp., and *P. palmata*. Other studied species include *Caulerpa* spp., *Porphyra dioca*, and *Ulva* spp. [185,186,187]. Recently, *S. platensis* and its proteins and peptides have been shown to possess antidiabetic activity. Ou et al. [188] reported that phycocyanin from *Spirulina platensis* alleviates alloxan-induced diabetes in mice by activating the insulin signaling pathway and enhancing glucokinase expression in the pancreas and liver. Sadek et al. [189] demonstrated that *S. platensis* prevents hyperglycemia in rats by modulating gluconeogenesis and apoptosis. Similarly, Aissaoui et al. [190] found that *S. platensis* reduced blood glucose levels by 79% compared with the control. Furthermore, the antidiabetic peptide LRSELAAWSR, identified from *Spirulina platensis*, exhibited inhibitory effects on α-glucosidase (IC_50_ = 134.2 μg/mL) and DPP-IV (IC_50_ = 167.3 μg/mL), with moderate activity against α-amylase (IC_50_ = 313.6 μg/mL). The enzymatic hydrolysate from *Palmaria palmate*, containing peptides Ile-Leu-Ala-Pro, Leu-Leu-Ala-Pro, and Met-Ala-Gly-Val-Asp-His-Ile, has been showing DPP-IV inhibitory activity with IC_50_ values in the range of 43–159 μM [185]. In addition to marine algae, peptides isolated from fish have also demonstrated antidiabetic properties. For instance, *Capros aper* has shown potential as a source of antidiabetic peptides, with the peptides IPV and IPVDM, produced through enzymatic hydrolysis, exhibiting in vitro DPP-IV inhibitory activity with IC_50_ values of 5.61 and 21.72 μM, respectively [191].

Proteins derived from mollusks have shown promising antidiabetic properties. Notably, protein hydrolysate from blue mussels (*Mytilus edulis*) has demonstrated the ability to inhibit dipeptidyl peptidase-IV (DPP-IV), an enzyme (IC_50_ range of 0.33–2.43 mg/mL) in glucose metabolism [192]. The antidiabetic effects of the sea cucumber (*Holothuria nobilis*) have been reported, mainly from hydrolysates extracted from low-edible-value species. These hydrolysates exhibit hypoglycemic, hypolipidemic, and insulin-sensitizing properties by activating the PI3K/Akt signaling pathway. Notably, aliphatic amino acid-containing peptides may play a key role in improving insulin resistance [59]. An enzymatic hydrolysate fraction from sea cucumber (*Stichopus japonicus*) has demonstrated significant antidiabetic potential. It enhances glucose uptake dose-dependently in high-insulin-induced insulin-resistant HepG2 cells and 3T3-L1 adipocytes. Additionally, it exhibits vigorous DPP-IV inhibitory activity, reaching 72.01% at 1 mg/mL, with an IC_50_ of 0.52 mg/mL [38].

Harnedy et al. [193] demonstrated that protein hydrolysates derived from Atlantic salmon (*Salmo salar*) skin gelatin and trimmings exhibit potential in vitro antidiabetic properties. Specifically, these hydrolysates were found to stimulate the secretion of glucagon-like peptide-1 (GLP-1), a key incretin hormone that enhances insulin release, thereby contributing to glucose homeostasis. In addition, Rivero-Pino et al. [84] reported that peptides from *Sardina pilchardus* protein hydrolysates inhibited DPP-IV, a key enzyme in diabetes regulation. Peptides (<1400 Da) obtained via enzymatic hydrolysis and size exclusion chromatography (SEC) purification, particularly those with NAPNPR and YACSVR sequences, were identified as contributors to this activity.

Thus, the findings above underscore the potential of marine-derived proteins and bioactive peptides as promising candidates for designing novel antidiabetic therapeutics.

### 4.10. Anticoagulation Potential

Thrombin plays a crucial role in coagulation by transforming soluble fibrinogen into insoluble fibrin, leading to clot formation. The coagulation cascade consists of intrinsic and extrinsic pathways, both linking at factor Xa formation, which, along with factor Va, forms prothrombinase to generate thrombin. Excessive thrombin activity can result in thrombosis, contributing to cardiovascular diseases like stroke and deep vein thrombosis. Anticoagulants target key factors in this cascade, such as thrombin and factor Xa, to prevent excessive clotting and reduce the risk of life-threatening cardiovascular events [131,194,195]. Marine organisms are gaining attention for their novel bioactive compounds, driving increased research into marine bioactive peptides and their therapeutic potential. Recently, many anticoagulant peptides derived from marine organisms have been studied. Indumathi and Mehta [196] identified an anticoagulant peptide (NMEKGSSSVVSSRMKQ) from the edible seaweed *Porphyra yezoensis*, which significantly extended the activated partial thromboplastin time (APTT) from 35 s to 320 s, with an IC_50_ value of 0.3 μM. A pepsin-derived anticoagulant peptide (DFEEIPEEYLQ, 1264.36 Da) from the oyster (*Crassostrea gigas*) inhibited thrombin by competing for anion-binding exosite I. The mechanism involved strong binding affinities and prolonged clotting through hydrogen-bonding, electrostatic, and hydrophobic interactions [197]. Similarly, the anticoagulant dodecapeptide (IEELEEELEAER) from the oyster (*Crassostrea gigas*) exerts its effect by inhibiting FXa and thrombin activity, crucial enzymes in the coagulation cascade. It further disrupts clot formation by blocking factor X activation through intrinsic and extrinsic tenase complexes and inhibiting prothrombin activation via prothrombinase complexes. Beyond its role in coagulation, the same peptide targets human umbilical vein endothelial cells (HUVECs), which play a protective role in vascular health. It restores thrombin-mediated barrier dysfunction, preserving endothelial integrity, and modulates the endothelial antithrombotic phenotype, contributing to a reduced pro-coagulant state [198,199]. A trypsin-derived hydrolysate from *Mytilus edulis* showed antithrombotic activity, with salt- and acid-soluble fractions showing the highest effects (85.74 and 82.00%, respectively, at 5 mg/mL). Molecular docking analysis revealed that the active peptide (ELEDSLDSER) interacts with thrombin through amino acid binding sites, contributing to its inhibitory mechanism [200]. Peptides from the adductor muscle of *Mytilus edulis* (VQQELEDAEERADSAEGSLQK, RMEADIAAMQSDLDDALNGQR, and AAFLLGVNSNDLLK) exhibited anticoagulant activity by inhibiting coagulation factors through molecular interactions [201]. Cheng et al. [202] reported the starfish anticoagulant protein (7.5 kDa) from *Acanthaster planci* disrupts blood coagulation by prolonging aPTT and PT, indicating its strong anticoagulant potential. It works by inhibiting factor X activation through intrinsic and extrinsic tenase complexes and blocking prothrombin activation via prothrombinase complexes. This dual-action mechanism prevents thrombin formation, effectively slowing clot development. Furthermore, the horseshoe crab-derived peptide tachyplesin I (KWCFRVCYRGICYRRCR) exhibits potent anticoagulant properties by prolonging bleeding and clotting time and reducing thrombosis in a carrageenan-induced tail thrombosis model. It interferes with thrombin activity and inhibits thrombin-induced platelet aggregation by disrupting the PI3K/AKT signaling pathway, a crucial mechanism in platelet activation [165]. In addition to other marine sources, marine bacteria can also serve as potential producers of anticoagulants. For example, the marine bacterium (*Oceanimonas* sp. BPMS22) produces an anticoagulant protein (30.0 kDa) that effectively inhibits plasma clot formation, likely interfering with key coagulation factors [203]. In addition, the marine bacterium *Bacillus velezensis* Z01 also produces a fibrinolytic protein (32.3 kDa) with potent anticoagulant properties. It ameliorates blood coagulation by prolonging aPTT, PT, and TT, indicating its interference with coagulation factors. Additionally, it degrades fibrin and fibrinogen, promoting fibrinolysis, and it blocks platelet aggregation, further preventing clot formation [204]. The fibrinolytic protein (50 kDa) from the marine bacterium *Pseudomonas aeruginosa* KU1 shows high bradykinin-binding affinity. It demonstrates thrombolytic activity in a carrageenan-induced murine tail thrombosis model, showcasing its potential as a marine-derived clot-dissolving agent [205]. *Streptomyces radiopugnans* VITSD8, isolated from marine brown tube sponges (*Agelas conifera*), produces a fibrinolytic protein capable of breaking down fibrin—similarly, *Serratia marcescens* and *Fictibacillus* sp. SKA27 produce fibrinolytic proteins exhibiting fibrin degradation activity [206,207,208].

Marine microalgae have potential for thrombosis treatment. For example, the fibrinolytic enzyme (protein) from *Chlorella vulgaris*, a metal-dependent serine protease, degrades fibrin and lyses 25.6% of thrombi in vitro within 90 min, with minimal red blood cell lysis (<4%), making it a promising thrombolytic agent [209]. The phycocyanin protein from Spirulina (*Arthrospira maxima*), a blue-green alga, exhibits anticoagulant properties by inhibiting the platelet-activating factor (PAF) and thrombin-induced platelet aggregation, preventing excessive clot formation [210].

Despite growing attention to anticoagulant peptides from marine origin, there are fundamental gaps. The precise molecular mechanisms, bioavailability, stability, and metabolic fate of these peptides should be explored. Most studies are limited to in vitro and computational approaches, with minimal in vivo or clinical validation. Further optimization of peptides for potency and specificity and examination of possible synergism with existing therapies is pending. These gaps must be filled with mechanistic research, pharmacokinetics, and clinical trials to advance marine-derived compounds as anticoagulant therapeutic agents.

### 4.11. Miscellaneous 

Unhealthy lifestyles, societal pressures, and aging contribute to the rising majority of memory dysfunction. Additionally, the risk of neurodegenerative disorders like Alzheimer’s and Parkinson’s is rising due to progressive neuronal damage and neuropathy. Marine-derived proteins and peptides have gained significant attention for their neuroprotective effects and their potential as effective, natural treatment strategies against neurodegeneration and cognitive decline. For example, Zhao et al. [211] reported that NDEELNK, a peptide from the sea cucumber ovum, exerted neuroprotective effects by enhancing cellular energy metabolism, regulating the cholinergic system, and activating the PKA/BDNF/NGF signaling pathway to protect against PC12 cell damage. Similarly, the sea cucumber (*Stichopus japonicas*)-derived peptide FYDWPK improved cognitive and memory function in a scopolamine-induced neurotoxicity model. It alleviated learning deficits, mitigated cholinergic dysfunction, and regulated oxidative balance, reducing pathological alterations in dementia-induced mice [212]. Furthermore, a sea cucumber-derived hydrolysate has demonstrated neuroprotective potential by enhancing behavioral performance, restoring cholinergic function, and preventing hippocampal nerve cell apoptosis in scopolamine-induced cognitive impairment in Kunming mice [14]. Moreover, the shrimp-derived peptides QMDDQ and KMDDQ enhance neuroprotection by increasing acetylcholine (ACh) and hindering acetylcholinesterase (AChE), with QMDDQ showing exceptional efficacy. Its N-terminal glutamine helps AChE interaction, supports energy metabolism, and activates the PKA/CREB/BDNF pathway. In amnesic mice, QMDDQ improved memory, increased hippocampal ACh, and reduced AChE activity, highlighting its therapeutic potential [213].

The osteogenic potential of the sea cucumber (*Stichopus japonicus*) hydrolysate has been demonstrated through its ability to promote bone formation and regeneration. It enhances proliferation, migration, differentiation, and mineralization in mouse preosteoblasts (MC3T3-E1) by activating the WNT/β-catenin and BMP/MAPK signaling pathways. Additionally, it increases bone mineral density and mitigates bone loss in ovariectomized mice with estrogen deficiency, highlighting its potential as a natural therapeutic for bone health [214].

The antiaging properties of sea cucumbers as well as isolated proteins and peptides have been reported. For example, sea cucumber (*Cucumaria frondosa*) hydrolysate (CFH) contains low-molecular-weight (<3kDa) peptides that enhance antiaging effects. An in vivo study showed that CFH prolonged the lifespan of fruit flies and improved cognitive function in d-galactose-induced aging mice. Its protective mechanism involves upregulating Klotho, increasing antioxidant enzyme activity, reducing oxidative damage, and inhibiting acetylcholinesterase, making CFH a potential antiaging ingredient [215]. Similarly, a hydrolysate fraction from the sea cucumber (*Stichopus variegates*) has been shown to promote longevity in normal and D-galactose-induced aging fruit flies. Additionally, it attenuated oxidative injury in Kunming mice subjected to D-galactose-induced aging [216]. 

A novel peptide isolated from the skin hydrolysates of cod (*Gadus macrocephalus*) inhibits the neuraminidase activity of the influenza A H1N1 virus. Acting as a neuraminidase blocker, it effectively suppresses viral infection (influenza A virus) in Madin–Darby canine kidney (MDCK) cells, suggesting its potential for influenza treatment [217].

Studies have shown that peptides isolated from sea cucumbers exhibit antifatigue effects by enhancing exercise performance and delaying physical exhaustion. Two peptides, SCP-1 (lower degree of hydrolysis) and SCP-2 (higher degree of hydrolysis), were identified that improved exercise endurance. SCP-2 demonstrated more muscular antifatigue activity than SCP-1, mediated through the NRF2/ARE and AMPK/PGC-1α pathways [102]. Similarly, a hydrolysate fraction from *Stichopus japonicus* enhanced mitochondrial quality, supporting energy homeostasis, regulating gluconeogenesis and fat metabolism, and boosting antioxidant capacity, resulting in improved antifatigue properties in C57BL/6J mice [86].

An enzymatic hydrolysate derived from Atlantic salmon (*Salmo salar*) viscera using pepsin has demonstrated anti-allergic properties. The identified peptide sequence, Thr-Pro-Glu-Val-His-Ile-Ala-Val-Aso-Lys-Phe, effectively inhibits β-hexosaminidase release during IgE-mediated degranulation of RBL-2H3 cells, highlighting its potential as a natural anti-allergic agent [59].

Appetite-regulating proteins and peptides have been identified from various marine sources. Cholecystokinin (CCK) and glucagon-like peptide-1 (GLP-1) are key hormones involved in appetite control and satiety, serving as valuable biomarkers for evaluating satiety responses in vivo and in vitro. Several studies have demonstrated the potential of marine-derived peptides to influence CCK production. Notably, a low-molecular-weight peptide (1.0–1.5 kDa) from shrimp head protein hydrolysate significantly stimulated CCK release from gut endocrine cells [218]. Wang et al. [53] reported that tilapia skin gelatin hydrolysate enhanced GLP-1 secretion in streptozotocin-induced diabetic rats after 30 days of treatment, an effect attributed to its amino acid composition, highlighting the role of bioactive components in satiety regulation. Similarly, Nobile et al. [219] found that enzymatically hydrolyzed blue whiting muscle hydrolysate improved body composition and increased CCK and GLP-1 levels in mildly overweight individuals, with both 1.4 g and 2.8 g doses proving adequate. Cudennec et al. [220] also investigated *Sepia officinalis* viscera hydrolysates and observed significant stimulatory effects on CCK and GLP-1 secretion in STC-1 cells following simulated digestion. Furthermore, the consumption of algal biomass and protein concentrates from *Chlorella vulgaris* and *Pyropia seriata* (nori) was studied for the effects on postprandial metabolism and satiety. Both exhibited palatability and contributed to satiety regulation, influencing appetite-related hormones such as GLP-1 [221].

Sexual and reproductive health issues, including sexual dysfunction and infertility, are prevalent and involve millions of individuals across the globe. Data and meager evidence suggest that bioactive compounds, including proteins and peptides of marine organisms such as seahorses, oysters, and sea cucumbers, can substantially alleviate hormonal imbalance, infertility, sexual dysfunctions, and impotence. For example, a peptide extract from an autoclaved and ultrasonicated extract of *Urechis unicinctus* has been reported to improve erectile dysfunction in streptozotocin-induced diabetic rats (via oral administration) by enhancing NO/cGMP signaling and upregulating the transcription and translation of cavernosal nNOS and eNOS in penile tissues [222]. In addition, a commercial oyster peptide hydrolysate (source unknown) has been reported to enhance the hypothalamus–pituitary–gonad axis function in male Sprague-Dawley rats under heavy-load training while also reducing oxidative stress in the testis by upregulating steroidogenic enzyme expression, promoting spermatogenesis and steroidogenesis [223]. Similarly, a commercial oyster peptide sample (source unknown) was reported to effectively increase blood androgen levels in cyclophosphamide-induced male rats, reduce oxidative stress and pathological damage in the kidneys, and improve kidney and testicular health, positively impacting reproductive capacity [153]. Like peptides, marine-derived protein hydrolysates have been shown to have an ameliorative effect on reproductive dysfunction. For example, a protein hydrolysate obtained by alcalde from the seahorse (*Hippocampus kuda*) has been reported to improve reproductive dysfunction by increasing testosterone levels, follicle-stimulating hormone, luteinizing hormone, sperm count, and motility in streptozotocin-induced diabetic rats fed with a high-fat diet [224].

**Table 2:** Bioactivities of marine-derived proteins and peptides.

Bioactivity	Marine Source	Method of Extraction	Protein/Peptide	Mode of Action	Reference
Antioxidant	Skipjack tuna (Katsuwonus pelamis) and SkinSkipjack Tuna (Katsuwonus pelamis) Roe	Enzymatic hydrolysis Acid and enzyme hyrolysis	GHHAAA, PHPR, SVTEV, VRDQY, and SMDV Gelatin fraction (Type I collagen) Cys-Gly-Arg	Exhibited significant protective effects on H_2_O_2_-induced oxidative damage in human umbilical vein endothelial cells (HUVECs) by enhancing cell viability and antioxidant enzyme activity. DPPH radical scavenging activity DPPH, ABTS and metal chelating action	[225,226,227]
	Siberian sturgeon (*Acipenserbaerii*) cartilage	Enzymatic hydrolysis	GEYGFE (700.70 Da), PSVSLT (602.67 Da), and IELFPGLP (942.12 Da)	DPPH and hydroxyl free radical scavenging activity.	[228]
	Antarctic krill (*Euphausia* *superba*)	Alcalase hydrolysis Pepsin and protease hydrolysis	SLPY, QYPPMQY, and EYEA LKPGN and LQP	Showed strong reducing power, protective effects against H_2_O_2_-induced plasmid DNA damage, and the capacity to inhibit lipid peroxidation. Protected Chang liver cells against H_2_O_2_-induced oxidative stress by scavenging excess ROS, increasing mitochondrial membrane potential, and decreasing DNA damage and MDA content.	[102,107]
	Seahorse (*Hippocampus* *abdominalis*)	Alcalase hydrolysis	HGSH (436.43 Da) and KGPSW (573.65 Da) IGTGIPGIW (457.26 Da)	Mitigated oxidative stress-induced damage in human umbilical vein endothelial cells (HUVECs). Peptides effectively regulated AAPH-induced ROS production in Vero cells.	[88,229]
	*Pinctada fucata*	Enzymatic hydrolysis and chemical synthesis Bio-fermentation method	Peptides	Exhibiting hydroxyl and superoxide radical-scavenging properties along with cellular antioxidant activity.	[230]
	Squid (*Loligo formosana*) head	Enzyme hydrolysis	Arg-Glu-Gly-Tyr-Phe-Lys	DPPH and ABTS scavenging activity.	[231]
	Atlantic sea cucumber (*Cucumaria* *frondosa*)	Enzymatic hydrolysis (Alcalase)	TEFHLL	Myeloperoxidase inhibition reduces oxidative stress in vitro.	[109]
	*Cucumaria frondosa*	Enzymatic hydrolysis (Alcalase, flavozyme, Corolase)	Protein hydrolysate	Hydroxyl radical scavenging activity, ferric reducing.	[74]
	Stripped weakfish by-products (skin and bone)	Alcalase and protamex hydrolysis	IELIEKPMGIF (1288.71 Da) RADLSRELEEISERL (1814.95 Da)	ABTS and hydroxyl radical scavenging activity.	[91]
	Miiuy croaker (*Miichthys miiuy*)	Enzymatic hydrolysis (papain)	FWKVV (612 Da)	DPPH, hydroxyl, and superoxide anion radical scavenging activity.	[232]
	Green algae (*Dunaliella salina*)	Ultrasound extraction followed by in vitro digestion	ILTKAAIEGK (1042 Da) IIYFQGK (867 Da) NDPSTVK (759 Da) TVRPPQR (852 Da)	DPPH radical scavenging activity.	[64]
	Sea cucumber (*Acaudina* *molpadioides*)	Neutrase-assisted microwave hydrolysis Microwave-assisted alkaline protease hydrolysis	Phe-Leu-Ala-Pro Collagen protein	DPPH radical scavenging activity. Reduces ROS levels in H_2_O_2_-damaged RAW264.7 cells.	[39,165]
	Abalone (*Haliotis discus hannai*)	Water hydrolysis	ATPGDEG (752 Da)	DPPH radical, hydroxyl, ROS scavenging activity.	[233]
	Red microalgae (*Palmaria palmata*)	Enzyme hydrolysis (Corolase PP)	Ser-Asp-Ile-Thr-Arg-Pro-Gly-Gly-Asn-Met	Oxygen radical absorbance capacity (ORAC) and ferric reducing antioxidant power (FRAP) activity.	[93]
	Shrimp shell discards	Enzymatic hydrolysis (Alcalase, trypsin, chymotrypsin, and pepsin)	Protein hydrolysate KLVRGSKS and LPKKCLSARPG	Exhibited radical scavenging and ferrous ion chelating abilities. DPPH and ABTS radical scavenging activities.	[89,106]
	Sea squirt (*Halocynthia roretzi*)	Enzymatic hydrolysis (pepsin, Protamex)	LEW, MTTL, and YYPYQL WLP and ISW	DPPH and ABTS+ radical scavenging activity, Fe^2+^ chelating activity, ORAC, and reducing power.	[92,234]
	By-product of serra Spanish mackerel (*Scomberomorus brasiliensis*)	Enzymatic hydrolysis	Protein hydrolysate	DPPH and ABTS radical scavenging activity, ferric reducing ability, iron chelating ability.	[235]
Antimicrobial	*Octopus minor*	--	GWLIRGAIHAGKAIHGLIHRRRH	Exhibited antifungal activity against Candida albicans.	[123]
	Half-Fin Anchovy (*Setipinna taty*)	--	FEDQLR, ALERTF, and RHPEYAVSVLLR	Peptides increased H_2_O_2_ production and K^+^ leakage, causing irreversible membrane damage.	[128]
	Horseshoe crab (*Limulus polyphemus*)	--	RRGCFRVCYRGFCFQRCR	Disrupts plasma membrane integrity, causing non-apoptotic cell death.	[127]
	*Chlorella vulgaris*	Enzymatic hydrolysis (pepsin)	Protein hydrolysate (62 kDa)	Inhibitory effect of 34.1% on *E. coli* growth.	[236]
	Mollusk (*Cenchritis muricatus*)	Solid-phase synthesis method	SRSELIVHQRLF	Fungistatic effect on *Candida albicans*.	[237]
Antihypertentsive	By-catch shrimp (*Oratosquilla woodmasoni*) waste	Enzymatic hydrolysis (thermolysin and pepsin)	Asn-Gly-Val-Ala-Ala (431 Da)	Produces ACE-I inhibition peptide that could be utilized as an antihypertentsive.	[238]
	Leathery sea squirt (*Styela clava*)	Enzymatic hydrolysis (pepsin)	LWHTH	Binds to the active site of ACE.	[239]
	Pearl oyster (*Pinctada fucata*) shell and pearl	Enzymatic hydrolysis (Nucleicin and Orientase 22 BF) trypsin	Gly-Val-Gly-Ser-Pro-Tyr (578.7 Da) KKCHFWPFPW	ACE inhibitory activity.	[90,221]
	Squid (*Todarodes pacificus*) skin	Enzyamtic hydrolysis (alkaline protease)	FHGLPAK, IIAPPERKY, RGLPAYE, and VPSDVEF	Binds to the ACE active site via hydrogen bonding.	[240]
	Mackerel (*Scomber japonicas*)	Enzyamtic hydrolysis (papain)	PLITT	ACE is inhibited by hydrogen bonding interaction.	[72]
	*Larimichthys* *crocea*	Enzymatic hydrolysis (papain, trypsin)	IPYADFK WAR and WQR	Peptides bind to active or non-central sites of ACE through hydrogen bonding.	[73,241]
	Monkfish (*Lophius litulon*) swim bladders	Acid and enzyme hydrolysis (Alcalase, neutrase)	SEGPK (516.5 Da), FDGPY (597.6 Da), and SPGPW (542.6 Da)	Angiotensin-I-converting enzyme (ACE) inhibitory activities.	[12]
	*Gracilariopsis chorda*	Enzymatic hydrolysis (thermolysin)	IDHY, LRY, LVVER	ACE inhibitory activity.	[75]
	*Arthrospira* *platensis*	Enzymatic hydrolysis (papain, ficin, pepsin, or Alcalase)	Protein hydrolysate	ACE inhibitory activity.	[77]
	*Laminaria digitata*	Enzymatic hydrolysis (Viscozyme)	YIGNNPAKGGLF and IGNNPAKGGL Protein hydrolysates	ACE inhibitory activity.	[242,243]
	*Isochrysis zhanjiangensis*	Enzymatic hydrolysis (trypsin)	ETT FEIHCC	Suppresses Ang II-induced inflammation and apoptosis through non-competitive ACE inhibition. Peptide inhibits ACE through a non-competitive binding mode.	[81,145]
	Blue mussels (*Mytilus edulis*)	Enzymatic hydrolysis	IK, YEGDP, WF, and SWISS	The inhibitory effect emerges from their strong interaction with the ACE active site via hydrogen bonding, electrostatic forces, and hydrophobic interactions.	[70]
	Shortfin scad (*Decapterus* *macrosoma*) waste	Alcalase hydrolysis	RGVGPVPAA (<3 kDa)	ACE inhibitory activity.	[244]
	*Ruditapes philippinarum*	Enzymatic hydrolysis (pepsin and pancreatin)	GRVSNCAA and TYLPVH	ACE inhibitory activity.	[107]
	Pufferfish (*Takifugu flavidus*) skin	Enzyme hydrolysis	PPLLFAAL (1 kDa)	Peptides exhibit strong binding affinity to angiotensin-converting enzyme (ACE).	[245]
	*Chlorella* *pyrenoidosa*	Enzyme hydrolysis (pepsin and trypsin)	FLKPLGSGK, LFVAEAIYK, and QIYTMGK	Peptides have potential interaction with ACE and block the enzyme.	[83]
	Atlantic salmon bone	Enzyme (trypsin) hydrolysis	Phe-Cys-Leu-Tyr-Glu- Leu-Ala-Arg	ACE inhibition occurs through hydrogen bond interactions between the peptide and the enzyme.	[246]
	Mesopelagic fish (*Maurolicus muelleri* and *Meganyctiphanes norvegica*)	Enzymatic hydrolysis	Protein hydrolysate	Acetylcholine esterase inhibition.	[247]
	*Mazzaella japonica*	Thermolysin	VDAHY, CPYDWV, SRIYNVKSNG, DFGVPGHEP, VSEGLD, YRD, SSNDYPI, GGPAT, TIMPHPR, YGDPDHY, and NLGN	ACE inhibition.	[248]
	Porphyra dioica	Enzyme hydrolysis	Thr-Tyr-Ile-Ala	ACE inhibitory activity.	[146]
	Spirulina (*Arthrospira platensis*)	Enzyme hydrolysis	IRDLDYY	Inhibits ACE through hydrophobic interactions and specific residue binding.	[5]
	Tilapia (*Oreochromis* *niloticus*) skin	--	Leu-Ser-Gly-Tyr-Gly-Pro	Reducing oxidative stress and alleviating endothelial damage.	[182]
	*Ulva intestinalis*	Enzymatic hydrolysis (trypsin)	FGMPLDR (834.41 Da), MELVLR (759.43 Da)	Peptide activity relied on hydrogen bonds and Zn(II) interactions with ACE.	[249]
	Cobia (*Rachycentron* *Canadum*) skin	Enzymatic hydrolysis (Protamex and protease N)	Trp-Ala-Ala, Ala-Trp-Trp, Ile-Trp-Trp, and Trp-Leu	ACE inhibitory activity.	[63]
	Deep-water pink shrimp (*Parapenaeus longirostris*) waste	Enzyme hydrolysis	SSSKAKKMP, HGEGGRSTHE, WLGHGGRPDHE, and WRMDIDGDIMISEQEAHQR Protein hydrolysate	ACE inhibitory activity.	[24,250]
	Bangia fuscopurpurea	Enzymatic hydrolysis (pepsin and trypsin)	ALLAGDPSVLEDR (1356 Da) VVGGTGPVDEWGIAGAR (1641 Da)	ACE inhibitory activity.	[251]
	*Stylotella aurantium*	Enzymatic hydrolysis (pepsin)	YR (337 Da) and IR (287 Da)	ACE inhibitory activity.	[252]
Antiinflammation	Red algae (*Amansia multifida*)	Chemical hydrolysis	Protein (lectin)	Multi-faceted anti-inflammatory response: reducing edema, suppressing pro-inflammatory cytokines, limiting neutrophil migration, and enhancing antioxidant defenses through elevated glutathione levels.	[253]
	Puffer fish (*Lagocephalus* *guentheri*)	Enzymatic hydrolysates (trypsin, Alcalase, and papain)	MEPLGQG (731 Da) and LLHA (453 Da) ESPVL (544 Da)	Reduced expression of cytokines, including TNF-α, iNOS, and COX-2. Suppressed cytokine expression.	[71,254]
	Sturgeon (*Acipenser schrenckii*) cartilage	Enzymatic hydrolysis (papain and pancreatin); ethanol, hot pressure	LTGP (386.43 Da), LLLE (486.59 Da), LLEL (486.59 Da), VGPAGPAGP (721.79 Da), Protein hydrolysate	Decreases NO and IL-6 production, enhances IL-10 secretion, and inhibits p38, ERK, and JNK phosphorylation. Reduced NO content and proinflammatory cytokine IL-6 levels by inhibiting MAPK signaling pathways.	[76,255]
	Sole fish (*Psettodes erumei*)	Enzymatic hydrolysis	MTQML (622 Da)	Suppresses NO and NF-κB complex expression while downregulating the NF-κB signaling cascade.	[256]
	Pearl oyster (*Pinctada* *martensii*)	Enzymatic hydrolysis (neutral protease)	TWP (402.19 Da), TAMY (484.19 Da), FPGA (390.19 Da)	Decreases NO and pro-inflammatory cytokines (TNF-α, IL-6, IL-1β) while enhancing IL-10 production.	[134]
	*Porphyridium* sp.	Enzymatic hydrolysis (Viscozyme and Alcalase)	GVDYVRFF (1002.11 Da), AIPAAPAAPAGPKLY (1407.65 Da), LIHADPPGVGL (1088.25 Da)	Inhibits COX-1 and COX-2.	[78]
	Baijiao sea bass (*Lateolabrax* *maculatus*)	Ultrasonication aided extraction	AADGPMKGILGY (1192.38 Da), DAPAPPSQLEHIRAA	Suppresses NO production in LPS-induced macrophages.	[137]
	Marine crab (*Charybdis* *natator*)	Enzymatic hydrolysis (Alcalase, trypsin, and papain)	ESPVL (544 Da)	Inhibits iNOS and COX-2 expression at higher doses in LPS-induced macrophages.	[254]
	Herring (Clupea harengus)	Enzymatic hydrolysis	IVPAS (485.56 Da)	Reduces iNOS activation.	[257]
	Green mussel (*Perna viridis*) foot	Enzymatic hydrolysis (Alcalase)	EGLLGDVF (849.43 Da)	Reduces pro-inflammatory cytokines, NO, and COX-2 activation.	[87]
	Sea cucumber (*Apostichopus japonicas, Acaudina* *leucoprocta*)	Enzymatic hydrolysis	GPSGRP (569.60 Da), GPAGPR (553.60 Da), PQGETGA (658.64 Da), GFDGPEGPR (930.95 Da)	Suppresses TNF-α, IL-1β, and IL-6 by inactivating the MAPK/NF-κB pathway in an LPS-induced liver injury model.	[136]
	Crab (Charybdis natator) leg muscle	Enzymatic hydrolysis (trypsin, Alcalase, and papain)	LGLGAAVL (713.5 Da)	Suppression of LPS-mediated induction of COX-2 in RAW264.7 macrophage cells.	[133]
	Sardine (*S. pilchardus*) by-products	Neutral protease	Protein hydrolysate	Hindering the inflammation regulatin in the endothelial cells	[142]
Anti-cancer	Cyclina sinensis	Ultrafiltration and chromatographic method	Ile-Leu-Tyr-Met-Pro (635.71 Da)	Induces anti-proliferative effects by promoting apoptosis by upregulating protein expression, attributed to the hydrophobic properties of the amino acids alanine (A) and leucine (L).	[159]
	Catfish (*Clarias gariepinus*)	--	HSDGIFTDSYSRYRKQMAVKKY-LAAVLGRRYRQRFRNK-NH2	Exhibited dose-dependent cytotoxic activity and inhibition of cancer cell proliferation.	[258]
	*Sinonovacula constricta*	Enzymatic hydrolysis	Leu-Pro-Gly-Pro (382.46 Da) and Asp-Tyr-Val-Pro (492.53 Da)	Inhibited cell growth and reduced cell populations through the induction of apoptosis.	[259]
	Nile tilapia	--	FIHHIIGGLFSAGKAIHRLIRRRRR	Induced cytotoxic activity, leading to cell death through necrosis.	[157]
	*Spirulina platensis*	Enzymatic hydrolysis	HVLSRAPR	Showed anti-proliferative activity against cancer cells while exhibiting low cytotoxicity toward normal cells.	[260]
Antiallergic	Atlantic salmon (*Salmo salar*) *viscera*	Enzymatic (pepsin) hydrolysis	Thr-Pro-Glu-Val-His-Ile-Ala-Val-Aso-Lys-Phe	Inhibition of β-hexosaminidase release during IgE-mediated degranulation of RBL-2H3 cells.	[59]
Antidiabetic	Mesopelagic fish (*Maurolicus muelleri* and *Meganyctiphanes norvegica*)	Enzymatic hydrolysis	Protein hydrolysate	DPP-IV inhibitory activity.	[247]
	*Sardine pilchardus*	Enzymatic hydrolysis (subtilisin, trypsin, and Flavourzyme)	NAPNPR, YACSVR	DPP-IV inhibitory activity.	[84]
	*Spirulina platensis*	--	VPMPNK, RNPFVFAPTLLTVAAR, and LRSELAAWSR	α-amylase, α-glucosidase, and DPP-IV inhibition activities.	[184]
	*Porphyra* spp.	Enzymatic hydrolysis	GGSK, ELS	Inhibited α-amylase activity.	[261]
Antiviral	*Cod Skin*	Hot-water hydrolysis	PGEKGPSGEAGTAGPPGTPGPQG (2163 Da)	Neuraminidase blocker to inhibit influenza A virus in MDCK cells.	[217]
Anticoagulation	Marine *Bacillus velezensis Z01*	--	Fibrinolytic protein	Ameliorated blood coagulation (aPTT, PT, TT, and FIB).	[204]
	Marine bacterium (*Pseudomonas aeruginosa KU1*)	--	Fibrinolytic protein (50 kDa)	- High-affinity binding to bradykinin. - Thrombolysis in the carrageenan-induced murine tail thrombolytic model.	[205]
	*Spirulina* (*Arthrospira maxima*)	--	Phycocyanin protein	Prevented PAF and thrombin-induced platelet aggregation.	[210]
	Seaweed (*Porphyra yezoensis*) Nori	Enzymatic hydrolysis (pepsin)	NMEKGSSSVVSSRM (1.77 KDa)	Prolongation of APTT.	[196]
	Mussel (*Mytilus edulis*)	Enzymatic hydrolysis	Protein hydrolysates, KNAENELGEVYVR	Inhibited thrombin activity.	[200,262]
			Protein hydrolysates, ELEDSLDSER	Hindered thrombin action toward fibrinogen.	
	Oyster (*Crassostrea gigas*)	Enzymatic hydrolysis (pepsin)	Protein hydrolysates, TARNEANVNIY	Prolongation aPTT and TT.	[35,198]
			Anticoagulant heptapeptides, NAESLRK	- Prolongation of fibrinogen clotting time, aPTT. - Reduction in the mortality of mice with pulmonary embolism induced by thrombin.	
Immunoregulator	*Stolephorus chinensis*	Enzymatic hydrolysis (pepsin, neutral protease, alkaline protease, trypsin, and papain)	YVMRF (715.4 Da)	Immunomodulatory activity.	[14]
	Giant Croaker (*Nibea japonica*)	Neutral protease	Protein hydrolysate	Hydrolysate promotes the immune system through cell-mediated immunity.	[86]
Antiobesity	Hammerhead shark (*Sphyrna mokarran*) skin	Enzymatic hydrolysis (pepsin and papain)	Collagen peptides	Attenuating high-fat diet–alcohol-induced hyperlipidemia.	[101]
	Ark shell	Protein hydrolysate	LLRLTDL and GYALPCDCL	Modulating adipocyte differentiation.	[169]
	*Miichthys miiuy* muscle	Chemically synthethized	VIAPW and IRWWW	Inhibition of oleic acid (OA)-induced lipid accumulation in HepG2 cells along with a reduction in intracellular TG and TC levels.	[59]
	*Spirulina platensis*	Enzymatic hydrolysis (trypsin, alcalase, pepsin, papain, and Protamex)	NALKCCHSCPA, LNNPSVCDCDCMMKAAR, NPVWKRK, and CANPHELPNK	Inhibiotory effect on T3-L1 preadipocyte proliferation.	[263]
	Skate (*Raja kenojei*) skin	--	Collagen peptide (1050 Da)	Suppression of fat accumulation and regulation of lipid metabolism.	[173]

## 5. Applications of Marine Bioactive Peptides and Proteins

Marine-derived proteins and peptides have garnered significant attention due to their exceptional properties, making them promising candidates for industrial applications in the food, pharmaceutical, cosmeceutical, and nutraceutical sectors. A summary of these applications is provided below.

### 5.1. Food Industry

The potential applications of marine-derived proteins and peptides in the food industry are driven by their ability to enhance texture and mechanical properties along with their notable antibacterial and antioxidant activities. Collagen and gelatin are marine-derived proteins extensively utilized as food additives in the food industry, primarily due to their gel-forming properties, film-forming ability, texture enhancement, and water-holding capacity. Gel-forming and water-holding capacity of marine collagen in meat products has been reported by enhancing different bond-forming and amino acid interactions with other proteins. For example, marine cod skin collagen enhances the formation of ionic, disulfide, and hydrogen bonds in surimi gel when incorporated at 9%. This results in a maximum gel strength of 554 g×cm and a water-holding capacity of 74.66%, likely due to the increased interaction between charged amino acids and surimi myofibrillar proteins [94,264]. Similarly, Sousa et al. [265] demonstrated replacing pork backfat with hydrolyzed collagen in Frankfurt-type sausages. They observed that a 50% collagen substitution enhanced aroma, protein profile, oxidative stability, texture properties, and water-holding capacity compared with sausages made from whole pork meat. Marine-derived collagen is gaining increasing attention for its potential as an emulsifier in various food products, including ice cream, chocolate, yogurt, and butter. For instance, collagen hydrolysates have been shown to preserve the emulsion properties of butter and chocolate sauce, significantly extending their shelf life [266]. In addition, Shori et al. [267] found that fish collagen in Codonopsis pilosula yogurt improved o-phthaldialdehyde peptide concentrations and scored higher in sensory evaluations. Furthermore, a milk-based food product, paneer, has been developed by incorporating collagen extracted from fish scales. The resulting paneer showed higher moisture and protein content, which contributed to improved sensory and textural attributes [268]. Collagen derived from marine sources is increasingly used in beverages due to its low viscosity, high solubility, and capacity to enhance nutritional and functional attributes. Additionally, its antioxidant activity contributes to shelf life extension. For instance, research on incorporating salmon scale ossein-derived collagen into chrysanthemum tea demonstrated its impact on sensory, antioxidant, and physicochemical characteristics over a six-month storage period. Findings showed that the collagen addition increased the α-amino group content, pH, turbidity, and browning index while improving the beverage’s antioxidant properties, extending its shelf life to six months at 30 °C [269]. Marine collagen, recognized due to its native triple-helical form and fibril networks, is widely employed in packaging meat, poultry, and seafood products. Its application as an artificial casing is particularly valued due to its sustainable, biodegradable, and biocompatible properties. Collagen-based packaging materials provide intense moisture and oxygen barriers while offering enhanced mechanical, sensory, and organoleptic properties. Bhuimbar et al. [270] developed collagen–chitosan antibacterial food packaging films using thick black ruff skin collagen and pomegranate peel extract, demonstrating effectiveness against water vapor permeability and foodborne pathogens such as *Bacillus saprophyticus* LNB 333F5, *Bacillus subtilis* NCIM 2635, *Salmonella typhi* NCIM 2501, and *Escherichia coli* NCIM 2832. Song et al. [271] investigated tilapia fish skin collagen for active food packaging applications. Results revealed that collagen/zein electrospun films with gallic acid effectively delayed tilapia muscle deterioration, prolonging its shelf life. An edible film was developed using collagen–lysozyme, with a formulation of 0.5% lysozyme and 4% collagen (*w*/*v*), showing the most effective protection against bacterial growth while preserving fresh salmon fillets (*Salmo salar*) [29]. Ben and Slimane [272] demonstrated that a biofilm made from *Mustelus mustelus* collagen and chitosan showed superior UV barrier properties and antioxidant activity compared with chitosan alone. This biofilm holds promise as a sustainable bioactive material for preserving nutraceutical products. 

Marine-derived proteins and peptides are increasingly being incorporated into various food products at an industrial scale due to their associated health benefits. For example, cod-derived proteins have been investigated for their potential to influence insulin secretion and directly regulate blood glucose levels [273]. Additionally, bioactive peptides derived from sardine, tuna, and shark have been reported to reduce blood pressure by inhibiting the angiotensin-converting enzyme [274]. Furthermore, peptides isolated from seaweed species demonstrate various functional properties, including antidiabetic, antioxidant, anti-inflammatory, anti-tumor, and anti-allergic activities. Moreover, protein-rich marine species such as crab, shrimp, prawn, and crawfish have been shown to contribute to renal disease prevention and support weight reduction by lowering total and LDL cholesterol levels [4]. The gastric enzyme obtained from fish has been investigated as a potential alternative to calf-derived rennet for cheese manufacturing. Research is being conducted on the fermentation of marine algae with probiotic bacteria to develop functional food products. In this approach, seaweed species such as *Saccharina latissima* and *Laminaria digitata* serve as the sole nutritional source for probiotic bacteria, facilitating the production of bioactive compounds with health-promoting properties [275]. The food industry is prioritizing the development of low-salt products to mitigate the risks associated with excessive salt intake such as hypertension and cardiovascular diseases. Seaweeds have been investigated as potential salt substitutes, showing the ability to maintain the organoleptic properties of conventional salted meat products while delivering a healthier option [275].

### 5.2. Pharmaceuticals

Marine proteins have drawn interest in pharmaceuticals for applications in tissue engineering, scaffold fabrication, drug delivery, encapsulation, and microcarrier development due to their distinct structural and biochemical properties. Neuroprotection helps safeguard the brain from damage and dysfunction caused by toxins, genetic factors, or aging, preserving nerve cells and overall brain health. Marine-derived biopolymers like alginate, gelatin, collagen, chitosan, and hyaluronic acid serve as microcarriers for neuroprotective molecules, protecting them from degradation and securing their pharmacological efficacy [276]. Fish-derived gelatin is widely employed in capsule manufacturing due to its strength, elasticity, and sealing properties. It is being investigated for oral drug delivery and active ingredient release. Based on strength, gelatin is classified as hard (for powders) and soft (for liquids). Non-gelling, non-hydrolyzed gelatin from cold-water fish is preferred for capsule manufacturing in pharmaceuticals [277]. Marine collagen enhances drug delivery by improving the solubility, stability, and controlled release of pharmaceuticals. Sourced from fish, seaweeds, sponges, and jellyfish, it is superior to mammalian collagen due to its easy extraction, water solubility, and excellent chemical and physical stability [20]. Marine collagen is widely used in pharmaceuticals for wound healing and tissue regeneration due to its strength, elasticity, and ability to support tissue repair. Its high biocompatibility minimizes immune reactions, making it safer than mammalian collagen for medical applications [278]. Unlike proteins, marine-peptide-rich drugs or derivatives are already available on the market, with several others currently undergoing clinical trials. Marine-based peptide drugs, such as ziconotide and brentuximab vedotin, have undergone clinical testing and received FDA approval for pharmaceutical applications [279]. Ziconotide is a native marine peptide (25 amino acids) first isolated from the marine mollusk *Conus magus*. It exerts analgesic effects by binding to the presynaptic N-type calcium channel α1B subunit, thereby inhibiting the release of neuromodulators and neurotransmitters. Brentuximab vedotin is a drug derived from dolastatin 10, a peptide composed of N, N-dimethyl Val-OH, L-valine, (3R,4S,5S)-dolaisoleucine, (2R,3R,4S)-dolaproine, and (S)-dolaphenine. Dolastatin 10 was initially isolated from *Dolabella auricularia*, a marine mollusk, and is produced by the symbiotic cyanobacterium *Caldora penicillate* [280]. Glembatumumab vedotin is an antibody–drug conjugate derived from dolastatin 10 that targets transmembrane glycoprotein NMB (GPNMB) in GPNMB-positive metastatic triple-negative breast cancer [281]. Plitidepsin is a depsipeptide currently in Phase III clinical trials that is isolated from the tunicate *Aplidium albicans*. It inhibits SARS-CoV-2 replication by targeting eukaryotic translation elongation factor 1A (eEF1A) [280,282]. Dermochlorella is an oligopeptide-rich extract, derived from *Chlorella* sp., known for its skin-protective properties. It is actively being explored as a cosmetic ingredient for its potential skin-firming and -toning agent applications [283]. Additionally, a review reported that Katsuobushi oligopeptide is a linear pentapeptide isolated from dried bonito (*Katsuobushi*) that has been found to contribute to lowering blood pressure (antihypertensive). Certain antihypertensive capsules marketed as nutraceuticals incorporate this peptide as one of their active components [2]. Table 3 summarizes a few marine-derived peptide products available in the market and undergoing clinical trials.

### 5.3. Cosmeceuticals

The cosmeceutical potential of marine-derived proteins and peptides is well known, emphasizing their usefulness in skin care. For example, collagen, a protein isolated from marine organisms, notably salmon and codfish skins, shows significant cosmeceutical potential due to its excellent water retention properties, making it an effective natural humectant and moisturizer. It is non-irritant, making it suitable for dermal applications [285]. The skin plays a key role as a barrier against environmental stressors, including UV radiation, which accelerates aging through oxidative stress driven by free radicals. While intrinsic (age) and extrinsic (UV exposure) factors contribute, melanin provides natural UV protection. Fish gelatin hydrolysates (proteins and peptides) protect the skin from UV-induced damage by preserving its structural integrity and maintaining lipid balance through their antioxidant properties [286]. UV radiation exposure reduces antioxidant enzymes, such as total superoxide dismutase (T-SOD), catalase (CAT), and glutathione peroxidase (GSH-Px), which are part of the skin’s endogenous defense system against oxidative stress. Polypeptides isolated from Pacific cod (Gadus macrocephalus) were studied in mice for their effects on photoaging and oxidative skin damage. The results showed that gelatin hydrolysates significantly enhanced the activity of T-SOD, CAT, and GSH-Px, reduced lipid peroxidation, and lowered inflammatory cytokine levels. These effects may be attributed to the inhibition of NF-κB expression by the hydrolyzed gelatin [287]. In addition, gelatin and its hydrolysates from salmon skin were evaluated for their protective effects on mouse skin. The results showed that these compounds reduced UV-induced damage by increasing T-SOD, CAT, and GSH-Px levels. Additionally, they enhanced immune function by elevating the thymus index and promoting collagen content as indicated by increased hydroxyproline levels in the skin [277].

### 5.4. Nutraceuticals

Marine protein, including collagen, is used widely as a food supplement for joint repair, enhancing lean muscle growth, faster recovery, and improved cardiovascular health. There is increasing demand for sports nutrition to confer these benefits, increasing in popularity among athletes and active individuals. Collagen has shown effectiveness in treating rheumatoid arthritis, with studies reporting significant reductions in pain, morning stiffness, and joint swelling [288]. In the United States, collagen is recommended as a dietary supplement for managing osteoarthritis in the hand, knee, and hip. Sardine scale collagen is widely used in sports nutrition products for athletes due to its rich amino acid composition in various forms, including powdered and liquid supplements, multicomponent bars, and chewable marmalade. Marine collagen peptides derived from fish waste have been utilized as a fortifier in biscuits, enhancing their protein and antioxidant contents [289,290].

## 6. Challenges and Future Direction

Despite the immense potential of marine-derived bioactive proteins and peptides, several challenges hinder their large-scale utilization and commercial applications. Conventional extraction methods, including solvent-based techniques with acetic acid treatment, are impractical for marine sponge collagens due to their insolubility. Alkaline solubilization affects the amino acid composition, the proteins’ secondary structure, and the solubility of fish protein isolates (FPIs), ultimately affecting their functional and therapeutic potential. Numerous studies have explored enzymatic extraction, showing significant potential for high collagen yield. However, the high cost of enzymes challenges the industrial-scale implementation of enzymatic processes. Although microbial fermentation is inexpensive, industrial exploitation for bioactive peptide production is impeded due to a low yield of peptide generation and a lack of specificity of peptide formation. Protein extraction from microalgae is challenging due to glycoside side chains and protein–polysaccharide linkages. Alkaline solutions with reducing agents like β-mercaptoethanol improve yield but limit food applications. Using food-grade alternatives such as N-acetyl-L-cysteine (NAC) ensures safety compliance. Purification of target peptides for laboratory-scale evaluations typically relies on traditional techniques such as membrane separation, dialysis, gel filtration, ion-exchange chromatography, and reverse-phase high-performance liquid chromatography (RP-HPLC). However, the recovery of active peptides from hydrolysates and fermented extracts remains challenging due to their low concentrations. Advanced separation and purification techniques like ultrafiltration and chromatography are costly and require optimization for large-scale production. Marine peptides are less allergenic than other bioactive compounds, with low or no toxicity. The toxicity and allergenicity of marine peptides should be assessed before adding them as active ingredients in functional foods and pharmaceuticals. Stability and bioavailability are significant concerns for proteins and peptides. Peptides can be degraded by processing, storage, and digestion, which destroys their functional activity. Thus, developing advanced nanotechnology-based approaches for encapsulating marine peptides into various nanostructures such as nanoemulsions, nanoliposomes, and polymeric nanoparticles is essential to improve their bioavailability and in vivo stability. Nonetheless, many of the existing techniques remain viable for niche market production.

Additionally, the assessment of the safety of nanoencapsulated marine peptides, particularly their fate and behavior in food complexes and the human body, is required for their successful application in functional foods and therapeutics. While the bioactivities of many marine peptides have been assessed, their precise molecular mechanisms, target interactions, and metabolic pathways remain inadequately understood. Bioinformatics and computational approaches such as molecular docking and in silico screening are now being employed to identify peptide sequences, but more precisely, artificial intelligence-driven models can facilitate the discovery of novel bioactive peptides and predict their functionality. However, the strategies for the future application of bioactive proteins and peptides should focus on human clinical trials and validation along with regulatory approval to develop novel functional food ingredients or novel drugs to treat various life-threatening diseases. 

## 7. Conclusions

Marine-derived proteins and peptides show remarkable bioactivity potential, making them invaluable for food, pharmaceutical, nutraceutical, and cosmeceutical applications. Their myriad biological actions, such as antioxidant, antihypertensive, antidiabetic, anticoagulant, antimicrobial, anti-inflammatory, antithrombotic, anti-cancer, and immunoregulatory effects, spotlight their therapeutic importance. Compared with terrestrial sources, marine-derived bioactive compounds offer advantages such as metabolic compatibility, fewer allergenic problems, and no religious constraints. Despite their potential, challenges remain in the efficient extraction, purification, and stabilization of marine proteins and peptides. Conventional extraction methods, including solvent extraction, enzymatic hydrolysis, and advanced techniques like microwave-assisted and supercritical fluid extraction, often confront high costs, energy consumption, protein denaturation, and scalability limitations. Overcoming these limitations requires advancements in green extraction technologies, nanotechnology-based delivery systems, and the optimization of processing conditions to enhance bioavailability and functional stability. Prospective research should focus on sustainable sourcing, innovative bioprocessing methods, and clinical confirmation to demonstrate the safety and efficacy of marine-derived proteins and peptides for human health applications. In addition, interdisciplinary approaches integrating biotechnology, bioinformatics, and material science can further expand their use in functional foods, drug development, and regenerative medicine. By addressing these challenges, marine-derived proteins and peptides can serve as sustainable and highly effective bioactive compounds, supporting innovations in health and wellness industries while promoting the sustainable utilization of marine resources.

## Figures and Tables

**Table 3:** Marine-derived peptide products available in the market and undergoing clinical trials.

Marine Source	Compound/Drug	Product Type	Application	Status/Clinical Trial	References
*Aplidium albicans*	Plitidepsin(Aplidin®)	Natural product	Antiviral(SARS-CoV-2)	Phase III	[280,283]
	Plitidepsin (Aplidin®)	Natural product	Advanced cancer	Phase III	[284]
*Dolabella auricularia*	Brentuximab vedotin	Derivative	Cancer treatment	FDA approved	[279]
*Conus magus*	Ziconotide	Natural product	Analgesic	FDA approved	[279]
Bonito (*Katsuobushi*)	Katsuobushioligopeptide	Natural product	Antihypertensive	Sold as nutraceutical	[2]
Fish hydrolysate	Nutripeptin®/Hydro MN Peptide®	Natural product	Postprandial blood glucose control	Sold as nutraceutical	[2]
*Chlorella vulgaris*	Dermochlorella®	Natural product	Skin toner andfirmer	Sold as skin care product	[283]

## Data Availability

No original experimental data were generated in this study.
